# Current Antithrombotic Treatments for Cardiovascular Diseases: A Comprehensive Review

**DOI:** 10.31083/j.rcm2508281

**Published:** 2024-08-08

**Authors:** Kristian Galanti, Mario Di Marino, Davide Mansour, Sabrina Testa, Davide Rossi, Claudio Scollo, Roberta Magnano, Laura Pezzi, Alberto D'Alleva, Daniele Forlani, Piergiusto Vitulli, Leonardo Paloscia, Fabrizio Ricci, Giulia Renda, Sabina Gallina, Massimo Di Marco

**Affiliations:** ^1^Department of Neuroscience, Imaging and Clinical Sciences, G. D'Annunzio University of Chieti-Pescara, 66100 Chieti, Italy; ^2^University Cardiology Division, Heart Department, “SS Annunziata” Polyclinic University Hospital, 66100 Chieti, Italy; ^3^Cardiology and ICCU Department, Santo Spirito Hospital, 65124 Pescara, Italy; ^4^Department of Clinical Sciences, Lund University, 21428 Malmö, Sweden

**Keywords:** antithrombotic treatments, anticoagulation therapy, antiplatelet therapy, ischemic risk, bleeding risk, risk-to-benefit ratio, cardiovascular diseases

## Abstract

Antithrombotic therapies (ATT) play a pivotal role in the management of cardiovascular diseases, aiming to prevent ischemic events while maintaining a delicate balance with the patient’s bleeding risk. Typically, ATT can be classified into antiplatelet and anticoagulant therapies. Their application spans a broad spectrum of cardiovascular conditions, ranging from ischemic heart disease to atrial fibrillation, encompassing venous thromboembolisms and innovative structural interventional cardiology procedures. The global burden of cardiovascular diseases is steadily increasing, often giving rise to overlapping clinical presentations. Accordingly, the adoption of combined pharmacological approaches becomes imperative, potentially disrupting the delicate equilibrium between ischemic and bleeding risk, thus leading to nuanced pharmacotherapeutic pathways. In this context, contemporary investigations strive to identify a convergence point that optimizes the duration of medical therapy while addressing the need for antithrombotic effects, especially in the context of ischemic heart disease. This review aims to comprehensively revisit the main antithrombotic strategies in cardiovascular diseases, with the intention of enhancing a systematic approach which is key for the effective clinical management of these patients. Also, the review will examine the most impactful studies that have established the groundwork for current scientific evidence, with acknowledgement of special populations. Finally, we will cast a gaze into the future of this dynamic and evolving research field, exploring forthcoming perspectives and advancements.

## 1. Introduction

Cardiovascular diseases (CVD) represent a leading cause of premature mortality and escalating public health care costs [1, 2]. Their prevalence is widespread, often associated with reduced survival, and continues to exhibit an increasing trend. The global CVD burden has nearly doubled from 271 million in 1990 to 523 million, nowadays [1]. Given the pivotal role of antithrombotic therapies (ATT) in managing these conditions, it becomes clear that understanding these therapies is key to optimal clinical management. The primary goal of ATT, often categorized into antiplatelet and anticoagulant treatments, is to prevent ischemic events while carefully balancing the inevitable bleeding risk for the treated patient. The determination of the risk-benefit ratio (RBR) is crucial in this context. Tools, such as the assessment of high bleeding risk (HBR) status, aid clinicians in evaluating the ischemic and bleeding risks of each patient, based on historical data and current clinical status [3]. The congestive heart failure, hypertension, age ≥75 (double), diabetes mellitus, prior stroke/transient ischemic attack (TIA)/thromboembolism (double), vascular disease, age, sex category (female gender) (${\mathrm{CHA}}_{2}$${\mathrm{DS}}_{2}$VASc) score is another widely used tool, particularly valuable in stratifying patients with atrial fibrillation to discern the necessity for anticoagulant treatment [4, 5]. These tools provide significant support for clinicians in managing patients with CVD, enabling tailored decision-making regarding the initiation, escalation, or de-escalation of antithrombotic therapy. Considering the extensive nature of this subject and its possible clinical and pharmacological ramifications, the focus of this review is delimited to a comprehensive examination of pivotal studies that have shaped the modern landscape of antithrombotic management in cardiovascular pathologies.

## 2. Primary Prevention Strategies

Primary prevention via antiplatelet therapy for CVDs is one of the most debated topics, considering that it still lacks a unanimous agreement among major cardiology societies worldwide.

### 2.1 Systematic Reviews and Meta-Analysis for Primary Prevention Strategies

A collaborative meta-analysis encompassed six major trials from 1988 to 2005, involving approximately 95,000 patients treated with low-dose aspirin, except for one trial (500 mg), or placebo. It revealed an annual reduction of 12% in major adverse cardiovascular events (MACE) in the aspirin group compared to the placebo group. Aspirin demonstrated a decrease in major coronary events, primarily driven by a reduction in non-fatal myocardial infarctions (MI), without influencing mortality due to coronary disease, from any form of stroke or vascular events. However, a remarkable increase in significant bleeding events was noted, including intracerebral haemorrhage, major gastrointestinal (GI) bleeding, and other extracranial bleeding [6]. In 2016, the U.S. Preventive Services Task Force analysed a total of 11 trials, highlighting a 22% reduction in MACE within the aspirin group [7]. This reduction was specifically associated with a decrease in non-fatal MI over the initial 5 years of treatment using a daily aspirin dosage of 75 to 100 mg. However, no recognizable benefit was observed with regard to a reduction in cardiovascular or all-cause mortality. Instead, there was a notable increase in significant extracranial bleeding events, particularly the GI ones. These studies had some limitations, including: unclear baseline stratification of cardiovascular risk, variations in baseline characteristics of the study populations, variable duration and dosage of aspirin, higher prevalence of cigarette smoking or the concurrent use of new antihypertensive drugs or statins. For these reasons, it was key to conduct subsequent trials with the additional aim of investigating specific populations at cardiovascular risk (Table 1, Ref. [8, 9, 10, 11]).

**Table 1.** **Major randomized control trials for antiplatelet treatment in primary prevention**.

RCTs	Population	Results
ASPREE [11]	≥70-year-old patients (or ≥65 years among blacks and hispanics in the United States) without CVD, dementia, or physical disabilities, to receive Aspirin 100 mg daily or placebo.	No change in disability-free survival over a period of 5 years.
		Higher rate of major hemorrhage than placebo.
ARRIVE [9]	≥55-year-old men or ≥60-year-old women with moderate cardiovascular risk, to receive Aspirin 100 mg daily or placebo.	Overall incidence rate of serious adverse events similar in both treatment groups. Significant increase in gastrointestinal bleeding events in the aspirin group.
ASCED [8]	15,480 participants with diabetes but no CVD of note.	Reduced risk of thrombosis.
		Increased incidence of major bleeding events was observed.
TIPS-3 [10]	5713 participants with elevated INTERHEART Risk Score, randomized to receive a polypill (statin + beta-blocker + angiotensin-converting enzyme inhibitor + thiazide diuretic) or placebo, aspirin (75 mg) or placebo daily, and vitamin D or placebo monthly.	Combined treatment with a polypill plus aspirin led to a lower incidence of cardiovascular events that did placebo among participants without cardiovascular disease who were at intermediate cardiovascular risk.

CVD, cardiovascular diseases; RCTs, randomized control trials.

### 2.2 ASPREE, ARRIVE, ASCED Controlled Randomized Trial

The ASPREE (Aspirin in Reducing Events in the Elderly) study was conducted by randomizing patients aged 70 or older (or ≥65 years among blacks and hispanics in the United States) without CVDs, dementia, or physical disabilities to receive either 100 mg of aspirin daily or a placebo. The primary endpoint was a composite of death, dementia, or persistent physical disability, and was found to be similar in both groups, leading to early study discontinuation (*p* = 0.79). The secondary endpoint of major bleeding occurred in 3.8% of participants in the aspirin group, compared to 2.8% in the placebo group (hazard ratio (HR) 1.38; 95% CI 1.18–1.62; *p* < 0.001). Fatal or non-fatal haemorrhagic stroke (including subarachnoid haemorrhage) occurred in 0.5% of cases in the aspirin group and 0.4% in the placebo group. As a consequence, the low-dose aspirin use in older individuals without CVDs did not prolong disability-free survival but significantly increased the rate of major bleeding [8]. This information should always be considered in the evaluation of RBR for antiplatelet therapy in this particular population. The ARRIVE (A Randomized Trial of Induction Versus Expectant Management) trial randomized ≥55-year-old men or ≥60-year-old women with moderate cardiovascular risk, to receive low-dose aspirin or placebo. Patients with a high risk of GI or other bleeding and with diabetes were excluded. The primary efficacy endpoint was a composite outcome of time to the first occurrence of cardiovascular death, MI, unstable angina, stroke, or TIA. The primary endpoint occurred in 4.29% of the aspirin group and 4.48% of the placebo group (HR 0.96; 95% CI 0.81–1.13; *p* = 0.6038). The overall incidence rate of serious adverse events was similar in both treatment groups, nonetheless, with a significant increase in mild GI bleeding events in the aspirin group (HR 2.11; 95% CI 1.36–3.28; *p* = 0.0007) [9]. A total of 15,480 participants with diabetes but unknown CVD were randomized to receive either 100 mg of aspirin daily or a placebo in the ASCED (A Study of Cardiovascular Events in Diabetes) trial. At a mean follow-up of 7.4 years, severe vascular events (MI, stroke or TIA, or death from any vascular cause) occurred in a significantly lower percentage of participants in the aspirin group compared to the placebo group (8.5% vs. 9.6%; HR 0.88; 95% CI 0.79–0.97; *p* = 0.01). While reducing the risk of thrombosis, an elevated incidence of major bleeding events was observed. These events manifested in 4.1% of participants in the aspirin group compared to 3.2% in the placebo group (HR 1.29; 95% CI 1.09–1.52; *p* = 0.003). GI bleedings were predominant, together with extracranial hemorrhages [8]. The US Preventive Services Task Force published a new meta-analysis, revealing that the use of low-dose aspirin was significantly associated with a reduction in cardiovascular events (major cardiovascular events, total MIs, and ischemic strokes), albeit without a significant reduction in CVD-related and all-cause mortality, confirming previous data [12]. Aspirin was found to be significantly associated with an increase in haemorrhagic events, including both intracranial and extracranial bleeding. Unfortunately, there are no effective means to reduce the risk of intracranial bleeding, apart from a thorough analysis of the RBR for each patient. GI haemorrhagic events are the major side effects associated with aspirin, and evidence has demonstrated that the co-administration of proton pump inhibitor drugs reduces their occurrence. For this reason, it is advisable to combine these drugs with aspirin [13].

### 2.3 Current Guidelines and Future Perspectives

The current guidelines of the European Society of Cardiology (ESC) are restrictive regarding the use of aspirin in primary prevention, which is only weakly recommended in diabetic patients and those with multiple cardiovascular risk factors and in the absence of clear contraindications (Class IIB, Level of Evidence A) [14]. Conversely, the American College of Cardiology and the American Heart Association guidelines have identified age as a discriminating factor: patients with high cardiovascular risk without an increased bleeding risk aged between 40 and 70 years might be considered for primary prevention, with low class of recommendation (Class IIB, Level of Evidence A) [15]. The US Preventive Services Task Force emphasizes the need for cardiologists to assess on a case-by-case basis the initiation of primary prevention treatment in patients with a cardiovascular risk equal to or greater than 10% over 10 years who do not have bleeding risk factors [12]. While not solely focused on antiplatelet therapy efficacy, the recent TIPS-3 study (The International Polycap Study 3) emphasized unclear advantages in cardiovascular mortality or event rates, except for stroke incidence; markedly conflicting results were observed for hemorrhagic safety outcomes. To note, this trial investigated a population at true intermediate cardiovascular risk (mean INTERHEART risk score 17.9) [10]. The divergence in guidelines reflects the paucity of robust evidences derived from trials and meta-analyses in the context of primary prevention, posing a notable challenge for clinical cardiologists. Decisions necessitate meticulous consideration on an individualized basis, with a keen focus on the RBR.

## 3. Chronic Coronary Syndrome

Chronic coronary syndrome (CCS), a stable manifestation of the coronary artery disease (CAD), shows different clinical manifestations with distinct prognostic and therapeutic implications. The classical presentation involves anginal pain and dyspnea.

### 3.1 Primary Prevention

In patients with CCS without a history of ACUTE CORONARY SYNDROME (ACS) or percutaneous coronary intervention (PCI), primary prevention therapy with aspirin receives a weak recommendation (Class IIb, Level of Evidence C) due to conflicting meta-analyses [6, 16]. The ongoing ASA-IN trial (NCT05347069) results may aid in complexity unraveling the benefit of this. The CHARISMA study showed that clopidogrel plus aspirin was not significantly more effective than aspirin alone [17]. Therefore, dual antiplatelet therapy (DAPT) is not indicated in this setting.

### 3.2 Long-Term Secondary Prevention

**Fig. 1.**
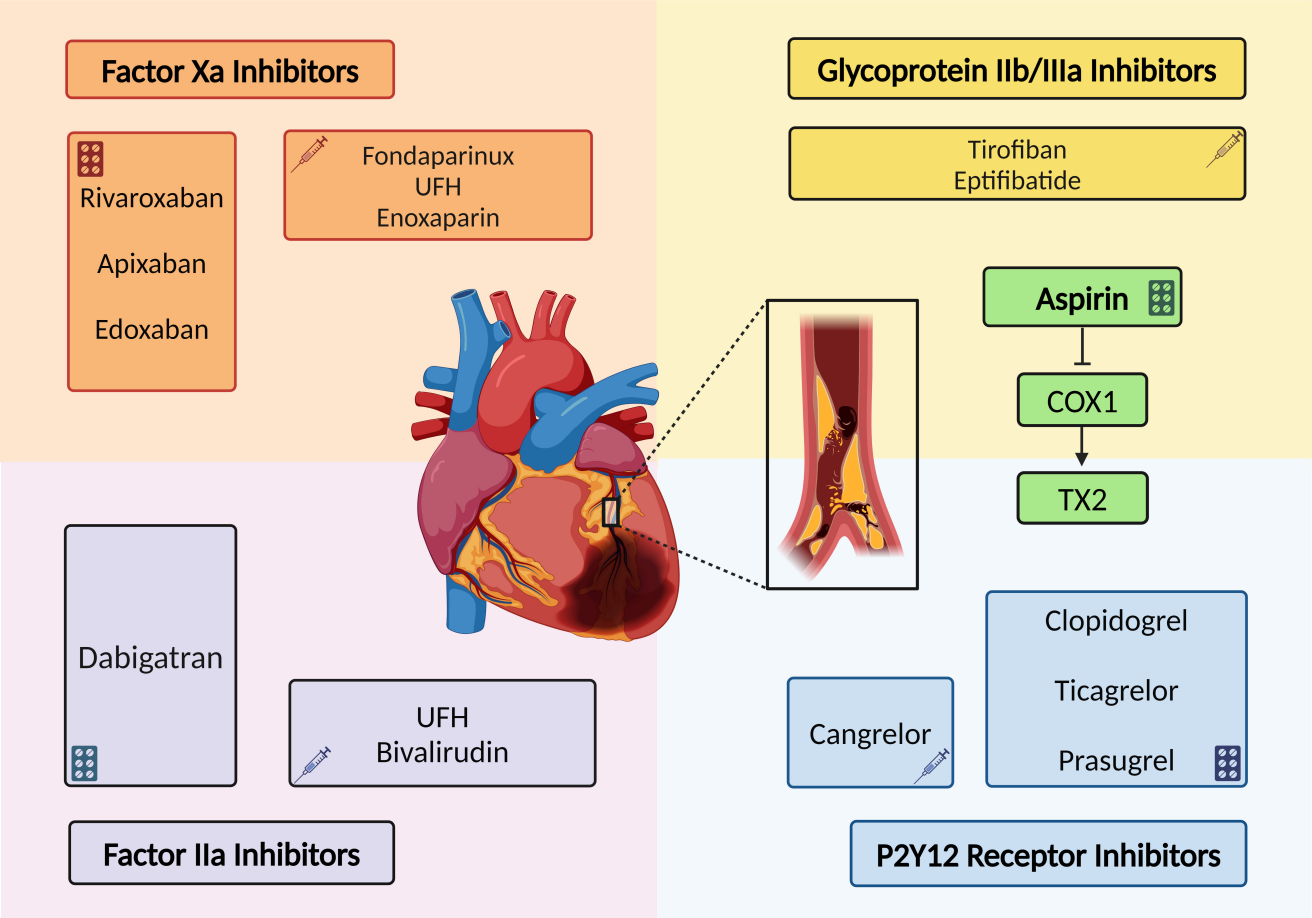
**Main classes of antithrombotic agents and their mechanism of action**. Antithrombotic therapies play a pivotal role in the management of major cardiovascular diseases, aiming to prevent ischemic events while maintaining a delicate balance with the patient’s bleeding risk. Typically, they can be classified into antiplatelet and anticoagulant therapies, both equally discernible in oral and parenteral ones. Given the steady increase in the global burden of cardiovascular diseases, the adoption of combined pharmacological approaches becomes imperative, always with the aim of preventing ischemic events while carefully balancing the inevitable bleeding risk for the treated patient. Factor IIa, activated coagulation factor II; Factor Xa, activated coagulation factor X; COX, cyclooxygenase; TX2, thromboxane A2; UFH, unfractioned heparin.

For long-term secondary prevention, aspirin is established as the cornerstone, and a 6-month DAPT regimen combining clopidogrel with aspirin is strongly recommended for CCS patients undergoing elective PCI [6]. This strategy is deemed optimal for achieving a balance between efficacy and safety across most patients [18, 19, 20, 21, 22, 23, 24, 25]. Bleeding risk influences therapeutic decisions, with a decreasing strength in the recommendation for high bleeding risk patients: a 3-month DAPT is suggested for individuals identified as high risk based on a PRECISE-DAPT Score ≥25 (Class IIa, Level A) [26, 27]. A shorter duration of DAPT is recommended at the lowest level of recommendation, as indicated by the results of two trials focused on specific types of drug-eluting stents (DES). However, these findings may not be automatically extrapolated to other contemporary DES [21, 22]. The use of Ticagrelor and Prasugrel in this clinical setting lacks sufficient data, limiting their use to specific high-risk situations (e.g., suboptimal stent deployment, complex left main stem, or multivessel stenting) or if DAPT cannot be employed due to aspirin intolerance (Class IIb, Level C). Therapeutic implications may arise from ongoing trials tailoring DAPT, using the latest-generation bioresorbable/biodegradable stents (SMART-CHOICEII, NCT03119012; PARTHENOPE, NCT04135989; TARGET DAPT, NCT03008083) or intracoronary imaging (OPTIMIZE-APT, NCT05418556). In patients with a history of ACS after 12 months, clopidogrel may be preferred as a default strategy in cases of aspirin intolerance (Class I, Level B) or in patients with concomitant peripheral arterial disease (PAD), history of stroke, or TIA (Class IIb, Level B), based on the results from CAPRIE [25]. Clopidogrel also shows equal efficacy to ticagrelor in symptomatic PAD (EUCLID study) [28]. A recent review and meta-analysis showed that short DAPT followed by P2Y12 inhibitor monotherapy reduces 1-year net adverse cardiovascular events (NACE) risk in complex PCI [29]. It is possible that in the future clopidogrel may become the default post-DAPT strategy. Additional evidence will emerge from ongoing SMART-CHOICE 3 trial (NCT 04418479). Yet, according to current evidence and ESC guidelines on ACS management, aspirin is the preferred antithrombotic agent following 12 months of DAPT. Additional therapeutic options for prolonging DAPT beyond 12 months depend on the balance between ischemic and bleeding risks. Fig. 1 shows the main classes of antithrombotic agents and their mechanism of action.

The PEGASUS TIMI 54 trial assessed two ticagrelor doses (60 mg or 90 mg) in post-myocardial infarction patients [30]. Both doses reduced the primary endpoint (cardiovascular death, MI, or stroke) by 15%, but increased clinically significant bleeding. The studied population, at high ischemic risk without recent bleeding or anticoagulation indications, may not represent all MI patients, especially those with different risk profiles [30]. The DAPT-Score is a valid tool that can help physicians understand those patients who can benefit from a longer DAPT duration after coronary stent placement. Caution is indicated for its use since only modest accuracy in derivation and validation cohorts was shown [31]. Factors for assessing ischemic and bleeding risks are listed in Table 2 (Ref. [30, 32, 33]), along with therapeutic regimens, drug indications, and respective recommendation classes according to ESC guidelines. A recent development in antithrombotic therapy for CCS is the possibility of using Non-vitamin K or direct oral anticoagulants (DOACs) in dual antithrombotic therapy (DAT) [34]. Specifically, the results of the COMPASS trial highlighted how the combination of rivaroxaban 2.5 mg and aspirin can reduce the composite endpoint of cardiovascular death, MI, or stroke, especially in patients with concomitant PAD [35]. Moreover, rivaroxaban at vascular dose plus aspirin may represent the only strategy for CAD patients without prior MI. The selection of a long-term secondary prevention strategy hinges significantly on the dynamic assessment of bleeding risk, a process that should be conducted at each follow-up. Notably, the literature reveals a diversity of criteria employed to define bleeding, thereby posing a challenge when attempting to compare various trials to generate high levels of evidence [36]. Table 3 (Ref. [30, 32, 33]) provides an overview of the main characteristics and outcomes of these randomized trials, accompanied by the definitions used for assessing bleeding risk.

**Table 2.** **Therapeutic options in patients with chronic coronary syndrome and history of acute coronary syndrome**.

Drug	Dose	Indication	References	Recommendation	
Clopidogrel	75 mg once daily	Post-MI in patient who have tolerated DAPT for 1 year	DAPT study [32]	IIa, A	IIb, A
Rivaroxaban	2.5 mg twice daily	Post-MI >1 year or multivessel CAD	COMPASS trial [33]	IIa, A	IIb, A
Ticagrelor	60 mg twice daily	Post-MI in patient who have tolerated DAPT for 1 year	PEGASUS-TIMI 54 trial [30]	IIa, A	IIb, A
Prasugrel	10 mg once daily or 5 mg once daily (if body weight <60 kg or age >75 years)	Post-PCI for MI in patients who have tolerated DAPT for 1 year	DAPT study [32]	IIa, A	IIb, A

■: high ischemic risk without high bleeding risk. ■: moderate ischemic risk without high bleeding risk. **High ischemic risk**: Diffuse multivessel CAD with at least one of the following: diabetes mellitus requiring medication, recurrent MI, PAD, or CKD with eGFR 1559 mL/min/1.73 $m^{2}$. **Moderate ischemic risk**: At least one of the following: multivessel/diffuse CAD, diabetes mellitus requiring medication, recurrent MI, PAD, HF, or CKD with eGFR 1559 mL/min/1.73 $m^{2}$. **High bleeding risk**: prior history of intracerebral haemorrhage or ischemic stroke, history of other intracranial pathology, recent gastrointestinal bleeding or anemia due to possible gastrointestinal blood loss, other gastrointestinal pathology associated with increased bleeding risk, liver failure, bleeding diathesis or coagulopathy, extreme old age or frailty, or renal failure requiring dialysis or with eGFR <15 mL/min/1.73 $m^{2}$. MI, myocardial infarctions; DAPT, dual antiplatelet therapy; CAD, coronary artery disease; PCI, percutaneous coronary intervention; PAD, peripheral arterial disease; CKD, chronic kidney disease; eGFR, estimated glomerular filtration rate; HF, heart failure.

**Table 3.** **Major RCTs and bleeding criteria to assess best long-term secondary prevention strategy in chronic coronary syndrome**.

RCTs	Study population	Primary endpoint	Main safety results	Bleeding criteria
DAPT [32]	Daily aspirin 75–162 mg + clopidogrel 75 mg or prasugrel 10 mg vs. daily aspirin 75–162 mg + placebo	Stent thrombosis 0.4% vs. 1.4%	Moderate or severe bleeds: 2.5% vs. 1.6%	GUSTO criteria and BARC criteria
		HR 0.29 [95% CI 0.17–0.48]	*p* = 0.001	
		*p * < 0.001		
PEGASUS [30]	(A) Ticagrelor 90 mg b.i.d. plus aspirin vs. (A’) Ticagrelor 60 mg plus aspirin vs. (B) placebo + aspirin	Composite of CV death, MI, stroke:	TIMI major bleeds: 2.6% in A vs. 2.3% in A’ vs. 1.06% in B (*p * < 0.001 for A or A’ vs. B)	TIMI bleeding classification
		A vs. B: HR 0.8 [95% CI 0.75–0.96]		
		*p* = 0.008;		
		A’ vs. B: HR 0.84 [95% CI 0.74–0.95]		
		*p* = 0.004		
COMPASS [33]	(A) Rivaroxaban 2.5 mg twice a day plus aspirin 100 mg once daily vs. (A’) Rivaroxaban 5 mg twice a day vs. (B) Aspirin 100 mg once daily	Composite of CV death, MI or stroke: 4.1% vs. 4.9% vs. 5.4% in A vs. A’ vs. B; *p * < 0.001 for A vs. B; *p* = 0.12 for A’ vs. B	Major bleeds A vs. B: 3.1% vs. 1.9%, HR 1.70 [95% CI 1.4–2.05]	Modified ISTH major bleeding
			*p * < 0.001	
			Fatal bleeds A or A’ vs. B: non-significant	
			Intracranial bleeds A vs. B: 0.3% vs. 0.3%, *p* = 0.60	

DAPT, dual antiplatelet therapy; HR, hazard ratio; CI, confidence interval; CV, cardiovascular; MI, myocardial infarction; ISTH, International Society on Thrombosis and Haemostasis; GUSTO, Global use of Streptokinase and t-PA for Occluded Coronary Arteries; BARC, Bleeding Academic Research Consortium; TIMI, thrombolysis in myocardial infarction.

### 3.3 Patients with Atrial Fibrillation

Antithrombotic therapy is crucial for patients with CCS and concomitant atrial fibrillation (AF), especially considering that 10–15% of AF patients undergo PCI for CAD [37]. Due to the absence of trials with a focus on CCS patients, there are few recommendations on the use of antiplatelet agents in the context of primary prevention for patients without a history of MI. Similarly, adding aspirin or clopidogrel to long-term DOAC-based therapy in CCS patients with concomitant AF and a history of MI not undergoing PCI has the lowest recommendation class [36]. This recommendation is contingent on a careful assessment of ischemic and bleeding risks and is based on the results of trials not originally designed for this purpose [35, 38]. In the context of patients undergoing PCI, the management becomes more intricate. For peri-procedural management, the discontinuation of anticoagulant therapy is not recommended when using vitamin K antagonists, while it is indicated when using DOACs. Pretreatment with aspirin and clopidogrel (Class I, Level C) is recommended, along with the use of intraprocedural unfractionated heparin (UFH) at a standard dose (reduced dose in case of vitamin K antagonists (VKA) use). Despite some variability, employing triple therapy after PCI, followed by randomization to DOAC and DAPT, demonstrated a notable reduction in major or clinically significant bleeding. Furthermore, it showed comparable rates of ischemic stroke and had a neutral effect on MACE and all-cause mortality compared to dual therapy [39, 40, 41, 42]. Subsequent meta-analyses have consistently affirmed a significant reduction in major bleeding with dual vs. triple and DOAC vs. VKA-based therapies, reporting similar stroke rates across all treatment arms [43, 44, 45, 46]. However, these analyses indicated higher rates of MI and stent thrombosis with dual vs. triple therapy. In particular, two meta-analyses demonstrated a statistically significant increase in stent thrombosis with dual vs. triple therapy. Consequently, after PCI in a patient with CCS and concomitant AF, the preference is to use DOAC rather than VKA (Class I, Level A). The duration of triple therapy is recommended for a period ranging from ≤1 week (Class I, Level A) to 6 months (Class IIa, Level C), contingent on the evaluation of bleeding and stent thrombosis risk. When choosing between ticagrelor, prasugrel and clopidogrel, it is key to consider that ticagrelor and prasugrel are associated with a higher risk of bleeding compared to clopidogrel, making their use weakly recommended as an alternative to triple therapy. Drawing on findings from the ISAR-TRIPLE and WOEST trials, it was established that the duration of dual therapy with DOAC or VKA and P2Y12 inhibitors should be 6 months, followed by continued anticoagulant therapy alone [47, 48]. For patients with an indication for VKA, due to the lower safety profile in terms of major or fatal bleeding, VKA dosing should be carefully regulated to achieve a target international normalized ratio (INR) of 2.0–2.5 and Time in Therapeutic Range (TTR) >70% [49]. **Supplementary Table 1** summarizes the most important randomized control trials (RCT) that have built the groundwork for antithrombotic management in CCS.

## 4. Acute Coronary Syndrome

ACS encompasses various conditions, including cases where individuals display recent changes in clinical symptoms or signs, regardless of whether there are associated modifications on a 12-lead electrocardiogram (ECG), and with or without acute rises in cardiac troponin (cTn) levels. MI is linked to the release of cTn and is determined in accordance to the fourth universal definition of MI. Individuals with suspected ACS are usually categorized according to their initial ECG findings, for the initial treatment and the subsequent management, in ST segment elevation MI (STEMI) and Non-ST segment elevation MI (NSTEMI). Unstable angina (UA) is defined as myocardial ischemia at rest or on minimal exertion in the absence of acute cardiomyocyte injury or necrosis [34].

### 4.1 Antithrombotic Therapy

ATT plays a central role in the treatment of ACS. According to the latest international guidelines, 12-month DAPT remains the cornerstone therapy for patients with ACS, both those managed medically and those undergoing PCI [34]. Several randomized controlled trials and meta-analyses have explored the possibility of shortening DAPT compared to the standard 12-month strategy and de-escalation strategies. In most cases, patients with a low to intermediate risk of ischemia were enrolled, and early monotherapy involved the use of either clopidogrel or ticagrelor. Some trials included a comparison with a control arm using more extended DAPT than the standard duration. Patients with STEMI were often excluded or underrepresented. The TWILIGHT trial investigated the impact of ticagrelor monotherapy compared to a combination of ticagrelor and aspirin for a duration of 1 year, following an initial 3-month-long DAPT involving ticagrelor and aspirin, specifically focusing on clinically relevant bleeding outcomes. Bleeding events (Bleeding Academic Research Consortium (BARC) type 2, 3, or 5 bleeding) were significantly reduced by omitting aspirin after 3 months, without a signal of increased ischaemic risk. STEMI patients were excluded from this trial [50]. The STOPDAPT-2-ACS trial explored the efficacy of a brief DAPT approach in patients with acute ACS [51]. The trial did not establish the non-inferiority of the investigational strategy for the composite endpoint of cardiovascular or bleeding events [51]. These findings suggest that a systematic approach of very short duration DAPT (i.e., <3 months) followed by clopidogrel monotherapy may not be a beneficial strategy for ACS patients. De-escalation refers to the shift from a potent P2Y12 receptor inhibitor class to clopidogrel. The TROPICAL-ACS trial demonstrated that the switch from prasugrel to clopidogrel after two weeks from the acute event, guided by platelet function, is not inferior to one year of DAPT with prasugrel in terms of net clinical benefit [52]. In the TOPIC trial, ticagrelor was also assessed alongside prasugrel for de-escalation, but without being guided by platelet function (or cytochrome P450 2C19 (CYP2C19) genotyping, as in the POPular Genetics trial); in these cases, it was observed that the de-escalation strategy reduced bleeding events, and the ischemic risk remained unchanged [53]. This data is crucial for emphasizing the importance of evaluating the response to clopidogrel, which, as is well-known, varies among patients [53]. In summary, the duration of DAPT can be shortened to three or six months, or even to one month, especially for HBR patients. De-escalation strategies can be initiated after a minimum of one month of DAPT with a potent P2Y12 inhibitor. Recent evidence supports the possibility of continuing antiplatelet therapy after DAPT with a P2Y12 inhibitor, rather than aspirin. This approach is an appealing option for clinical practice as it has been observed to reduce NACE at 1 year in patients undergoing PCI [29]. Cangrelor has been assessed in clinical trials for ACS during PCI. Trials such as CHAMPION and CHAMPION PHOENIX administered cangrelor either before or after PCI comparing it with clopidogrel. Considering its proven efficacy in preventing stent thrombosis in P2Y12 receptor inhibitor-naïve patients, cangrelor may be considered in these patients [54]. However, it is important to consider that this study not only included patients with ACS but also CCS patients.

### 4.2 Anticoagulant Therapy

Anticoagulation plays a pivotal role in the initial treatment of ACS and in the peri-procedural management of ACS patients undergoing an invasive strategy. UFH is the current default choice for anticoagulation in the acute setting [34]. Enoxaparin is a valid alternative to UFH: in a meta-analysis comparing these two molecules, there was no substantial difference in terms of mortality and major bleeding. Therefore, it is currently recommended, albeit with a lower level of evidence compared to UFH [55]. Bivalirudin is recommended as an alternative to UFH, with a lower recommendation class (IIa), particularly in patients with a working diagnosis of STEMI and heparin-induced thrombocytopenia [56]. In NSTE-ACS patients without an early invasive strategy, fondaparinux is recommended over enoxaparin, showing favorable outcomes in the OASIS-5 trial [57]. Based on the results of the OAIS-6 trial, fondaparinux is not recommended in patients with STEMI undergoing primary PCI (Table 4, Ref. [50, 51, 52, 53, 56, 57, 58, 59]). Triple antithrombotic therapy (TAT) in patients with ACS and an indication for anticoagulation poses a significant challenge for clinicians, navigating the delicate balance between thrombotic and bleeding risks. Literature suggests that opting for a shorter strategy (one week) results in lower bleeding risk without significantly increasing MACE risk compared to a longer strategy (one month) [60]. Fibrinolytic therapy, which is fundamental for STEMI patients unable to undergo prompt primary PCI, prevents 30 early deaths per 1000 patients treated within 6 hours from symptoms onset. The highest net treatment benefit refers to high-risk patients, including the eldest ones. Quick initiation, preferably within 10 minutes from initial diagnosis, is vital. Pre-hospital fibrinolysis, especially within 2 hours, reduces early mortality by 17% [61]. The STREAM trial advocated for pre-hospital fibrinolysis followed by early PCI, mirroring primary PCI for patients within 3 hours of symptom onset. Administering half the usual dose of tenecteplase reduces the risk of intracranial bleeding in patients aged over 75 [62, 63]. Patients treated with fibrinolysis, for whom an immediate invasive strategy via PCI is not feasible, should receive anticoagulation with low molecular weight heparin (LMWH) or UFH, with dosages adjusted for age and weight. This bridging therapy should be maintained until PCI is performed or for a maximum of 8 days, bearing in mind that PCI is recommended between 2 and 24 hours after the index event [34].

**Table 4.** **Major RCTs investigating the role of antithrombotic treatments in ACS settings**.

RCTs	Methods	Patients	Primary Endpoint
TWILIGHT [50]	Ticagrelor plus aspirin vs. ticagrelor plus placebo (alone) after three months of DAPT	7119	4% in ticagrelor plus placebo;
			7% in ticagrelor plus aspirin
STOPDAPT-2-ACS [51]	One to two months of DAPT followed by clopidogrel monotherapy vs DAPT one-year clopidogrel vs. aspirin	4169	3.2% in the 1-to 2-month DAPT;
			2.8% in the 12-month DAPT
TROPICAL-ACS-TRIAL [52]	Standard treatment with prasugrel for 12 months (control group) vs. a step-down regimen (1-week prasugrel followed by 1-week clopidogrel and PFT-guided maintenance therapy with clopidogrel or prasugrel from day 14 after hospital discharge; guided de-escalation group)	2610	7% of the guided de-escalation group;
			9% of the control group.
TOPIC TRIAL [53]	Standard treatment with aspirin and a newer P2Y12 blocker for one year vs. switch at one month to clopidogrel (unchanged DAPT vs. switched DAPT)	646	13.4% of the switched group;
			26.3% in the unchanged group.
POPular Genetics Trial [59]	Genotype-guided group vs. standard treatment group. First group without loss of function of *CYP2C19* received clopidogrel, those with loss of function prasugrel or ticagrelor.	2488	Primary bleeding outcome:
			9.8% of the genotype-guided group;
			12.5% of the standard treatment.
CHAMPION PHOENIX Trial [58]	Periprocedural administration of Cangrelor or clopidogrel, with either a 300- or 600-mg loading dose for the prevention of periprocedural complications in patients undergoing percutaneous coronary intervention	10,492	Cangrelor consistently reduced the primary endpoint in SA (stable angina) and ACS (odds ratio [OR]: 0.83 [95% confidence interval (CI): 0.67 to 1.01] and OR: 0.71 [95% CI: 0.52 to 0.96], respectively; interaction *p* = 0.41). Cangrelor also consistently reduced stent thrombosis in SA and ACS (OR: 0.55 [95% CI: 0.30 to 1.01] and OR: 0.67 [95% CI: 0.42 to 1.06], respectively; interaction *p* = 0.62).
OASIS-5 [57]	Patients with acute coronary syndromes received either fondaparinux (2.5 mg daily) or enoxaparin (1 mg per kilogram of body weight twice daily) for a mean of six days and evaluated death, myocardial infarction, or refractory ischemia at nine days (the primary outcome); major bleeding; and their combination. Patients were followed for up to six months	20,078	Primary-outcome events were similar in the two groups (579 with fondaparinux [5.8 %] vs. 573 with enoxaparin [5.7 %]; hazard ratio in the fondaparinux group, 1.01; 95% confidence interval, 0.90 to 1.13. The rate of major bleeding at nine days was markedly lower with fondaparinux than with enoxaparin (217 events [2.2 %] vs. 412 events [4.1 %]; hazard ratio, 0.52; *p* < 0.001).
OASIS-6 [56]	To evaluate the effect of fondaparinux, when initiated early and given for up to 8 days vs usual care (placebo in those in whom unfractionated heparin [UFH] is not indicated [stratum 1] or unfractionated heparin for up to 48 hours followed by placebo for up to 8 days [stratum 2]) in patients with STEMI.	12,092	Death or reinfarction at 30 days was significantly reduced from 677 (11.2%) of 6056 patients in the control group to 585 (9.7%) of 6036 patients in the fondaparinux group (hazard ratio [HR], 0.86; 95% confidence interval [CI], 0.77–0.96; *p* = 0.008). There was no benefit in those undergoing primary percutaneous coronary intervention. Significant benefits were observed in those receiving thrombolytic therapy (HR, 0.79; *p* = 0.003) and those not receiving any reperfusion therapy (HR, 0.80; *p* = 0.03).

RCTs, randomized controlled trials; DAPT, dual antiplatelet therapy; PFT, platelet function testing; ACS, acute coronary syndrome; STEMI, ST-segment elevation myocardial infarction; *CYP2C19*, cytochrome P450 2C19.

### 4.3 Future Perspectives

Recent evidence has delineated two phenotypes of atherosclerotic lesions that warrant attention: plaque rupture and plaque erosion [64]. In ACS patients, the characteristics and location of the plaque within the coronary vasculature influence platelet activation and thrombus composition. In-depth investigations into characterizing thrombus architecture are essential for identifying key pathophysiological factors, thus enhancing therapeutic efficacy in ACS patients. These models allow for the evaluation of novel platelet inhibitors (e.g., glycoprotein VI inhibitors) and/or anticoagulants, either as monotherapy or on top of the standard of care. Combining anti-inflammatory drugs with antithrombotic treatments holds promise in preventing cardiovascular atherothrombotic events, offering a potential avenue for ACS treatment (**Supplementary Table 2** summarizes ongoing RCTs on ACS).

## 5. Atrial Fibrillation

AF is a supraventricular arrhythmia marked by uncoordinated atrial electrical activity manifesting as irregularly irregular R-R intervals and the absence of distinct P waves on electrocardiography. The current estimated prevalence of AF in adults ranges from 2% to 4%, exhibiting an age-related increase [4]. Common AF symptoms include palpitations, dyspnea, shortness of breath and fatigue, with additional complaints such as chest pain, dizziness and syncope.

### 5.1 Patients Risk Stratification

AF is an independent risk factor for stroke, whose incidence can be reduced by using antithrombotic prophylaxis. However, thromboembolic risk is not homogeneous, depending on the presence of specific stroke risk factors or modifiers. Patients with moderate-to-severe mitral stenosis and mechanical prosthetic heart valves are considered at high risk of thromboembolism: for these patients, an ATT is strongly recommended. For all the other patients, common stroke risk factors are considered to stratify the risk, and these are summarized in the clinical risk-factor-based ${\mathrm{CHA}}_{2}$${\mathrm{DS}}_{2}$VASc score. This score demonstrates enhanced sensitivity in distinguishing lower and intermediate-risk patients, thereby refining therapeutic decision-making [65]. Current guidelines recommend oral anticoagulation for stroke prevention in AF patients with a ${\mathrm{CHA}}_{2}$${\mathrm{DS}}_{2}$VASc score ≥2 in men or ≥3 in women, and it should be considered in patients with a ${\mathrm{CHA}}_{2}$${\mathrm{DS}}_{2}$VASc score of 1 in men or 2 in women, based on net clinical benefit and consideration of patient values and preferences (Class of Recommendation I, level of Evidence A) [66]. However, alternative scores have been developed to assess thromboembolic risk. A recent systematic review and metanalysis analysed 19 scores and 76 updates, revealing that the ${\mathrm{CHA}}_{2}$${\mathrm{DS}}_{2}$VASc score showed inferior discriminative abilities compared with newer scores. Further external validations will be needed before considering novel scores in clinical practice [67]. Regarding bleeding risk, the HAS-BLED (Hypertension, Abnormal renal/liver function, Stroke, Bleeding history or predisposition, Labile INR, Elderly, Drugs/alcohol concomitantly) score has demonstrated superiority in assessing major bleeding risk in clinical practice, surpassing the performance of other risk scores. Therefore, careful consideration of the HAS-BLED score is necessary when estimating the RBR [68].

### 5.2 Main Therapeutic Option

Historically, coumarin derivatives have been the predominant therapeutic options for this condition, inhibiting several vitamin K-dependent coagulation factors (II, VII, IX, and X). However, the effectiveness and safety of VKAs hinge on the quality of anticoagulation control, contingent upon the maintenance of the INR within the therapeutic range (2.0 to 3.0). Furthermore, the metabolism of these drugs is closely linked to cytochrome P450 genetic polymorphisms and is also influenced by food and drug interactions, necessitating frequent monitoring and continuous daily dose adjustments. This complexity can ultimately lead to the risk of inadequate anticoagulation dosing [69]. The challenging manageability of warfarin has driven pharmacological research to explore new drugs, resulting in the approval of DOACs. These approvals are based on large RCTs and subsequent metanalyses (Table 5, Ref. [70, 71, 72, 73, 74]), demonstrating their better efficacy/safety profile compared with warfarin [70, 71, 72, 75]. Considering the phase III trials results and the better efficacy/safety profile of DOACs vs. VKAs, confirmed also by real world evidence, guidelines now recommend DOACs over VKAs for stroke prevention in AF patients who are eligible for anticoagulant therapy, excluding patients with moderate-to-severe mitral stenosis or mechanical heart valves (Class of Recommendation I, level of Evidence A) [76]. Thromboembolic risk is not equivalent for all forms of valvular heart disease (VHD) in patients with AF. Phase III clinical trials of DOACs included variable proportions of VHD patients and individually provided no evidence of a differential effect of DOACs over warfarin in patients with and without VHD [76, 77]. However, patients with moderate-to-severe mitral stenosis and mechanical heart valves were excluded from all phase III DOAC vs. warfarin trials in AF, due to their higher thromboembolic risk. Thereafter, clinical trials designed in these populations support the use of VKAs for these indications [73, 75, 78]. A possible explanation for the failure of DOACs in these settings is the direct inhibition of a single coagulation factor compared with warfarin which blocks the production of several factors of the intrinsic and common pathways, including factor IX (FIX), factor X (FX), and prothrombin, in addition to factor VII (FVII) in the extrinsic pathway, all playing a role in the thromboembolic mechanism related to mitral stenosis and mechanical prostheses.

**Table 5.** **Major RCTs for DOACs as a treatment option in AF**.

	ARISTOTLE [70]	AVERROES [74]	ROCKET-AF [72]	RELY-AF [73]	ENGAGE AF-TIMI 48 [71]
Study design	Randomized, double bind	Randomized, double bind, double dummy	Randomized, double bind, double dummy	Randomized, open label	Randomized, double bind, double dummy
Statistical objective	Non inferiority	Superiority	Non inferiority	Non inferiority	Non inferiority
Follow-up period	40 months	1.1 years	40 months	24 months	24 months
Primary efficacy	Composite of stroke and systemic embolism	Composite of stroke and systemic embolism	Composite of stroke and systemic embolism	Composite of stroke and systemic embolism	Composite of stroke and systemic embolism
Principal safety	Major bleeding	Major bleeding	Major bleeding	Major bleeding	Major and non-major clinically relevant bleeding
Warfarin arm	Dose-adjusted warfarin	ASA	Dose-adjusted warfarin	Dose-adjusted warfarin	Dose-adjusted warfarin
DOAC arm	Apixaban 5 mg BID, 2.5 mg if creatinine >1.5 mg/dL	Apixaban 5 mg BID, 2.5 mg if creatinine >1.5 mg/dL	Rivaroxaban 20 mg QD, 15 mg QD with CrCl 30–40 mL/min	Dabigatran 1150 or 110 mg BID	Edoxaban 60 mg or 30 mg QD
Inclusion	AF, flutter, stroke or 2 of: LVEF <40%, age >75, DM, HTN	AF, LVEF <35%, age >75, DM, HTN, previous stroke, PAD	AF, stroke or 2 of: LVEF <35%, age >75, DM, HTN	AF, stroke or 2 of: LVEF <35%, age >75, DM, HTN	AF, LVEF <35%, age >75, DM, HTN, previous stroke, CHADS >2
Exclusion	Intracranial bleed, stroke within 7 days, valvular heart disease, renal insufficiency, ASA and clopidogrel use	Serious bleed, stroke within 10 days, valvular heart disease, renal insufficiency, drug abuse	TIA within 3 days, stroke within 14 days, valvular heart disease, high bleeding risk, liver disease, kidney disease, aspirin use	Severe heart disease, stroke within 14 days, high bleeding risk, elevated creatinine, liver disease	Creatine clearance <30 mL/min, high bleeding risk, use of aspirin or clopidogrel, valvular heart disease, stroke within 30 days
Type of bleeding reported	Major bleeding, intracranial and GI	Major bleeding, intracranial and GI	Extracerebral, intracranial and major bleeding	GI, intracranial and major bleeding	Major bleeding
${\mathrm{CHADS}}_{2}$score	2.1	2.1	3.48	2.1	2.8

DOACs, direct oral anticoagulants; AF, atrial fibrillation; ASA, acetylsalicylic acid; BID, bis in die; QD, quaque die; CrCl, creatinine clearance; LVEF, left ventricular ejection fraction; DM, diabetes mellitus; HTN, hypertension; PAD, peripheral artery disease; TIA, transient ischemic attack; GI, gastrointestinal; RCT, randomized controlled trial.

### 5.3 Role of DOACs in Special Populations: Obesity and Renal Impairment

Table 6 outlines the most significant differences among DOACs in terms of mechanism of action and pharmacological characteristics: some of them are key for a tailored approach to clinical management. Apixaban exhibits the lowest renal excretion (27% renal elimination), while dabigatran undergoes almost complete renal elimination (80%). Dabigatran, being a pro-drug, requires activation by plasma and hepatic esterases, affecting both its bioavailability (6.5%) and absorption [79]. Additionally, the presence of tartaric acid increases the risk of dyspepsia and GI symptoms, thus leading to treatment interruptions at an incidence of up to 35.6%, compared to 1.6% and 0% for rivaroxaban and apixaban, respectively. Rivaroxaban, however, can be influenced by food, necessitating its intake shortly after meals. Certain special populations warrant particular attention in anticoagulation management and necessitate dosage adjustments. Obesity has implications for renal clearance, metabolism, and drug delivery. In individuals with obesity, both renal blood flow and renal clearance increase potentially diminishing anticoagulant activity [80]. The use of the Cockcroft-Gault (CG) formula, recommended by guidelines based on clinical trial results, may lead to an overestimation of renal function and misdiagnosis of hyperfiltration. Estimation of glomerular filtrate using formulas such as Modification of Diet in Renal Disease (MDRD) or Chronic Kidney Disease Epidemiology Collaboration (CKD-EPI) is advised [81]. Meta-analyses and reviews of trial-based publications focusing on body mass index (BMI) levels indicate that in patients with grade III obesity (BMI 40–49 kg/$m^{2}$), there is limited efficacy data for dabigatran and rivaroxaban, while data for edoxaban and apixaban are more robust. In individuals with large obesity (BMI >50 kg/$m^{2}$), data are limited for all DOACs and warfarin is recommended [82, 83]. The presence of chronic kidney disease (CKD) poses challenges for anticoagulation therapy in patients with AF, as it increases both thromboem bolic and bleeding risks. CKD and AF are interconnected conditions, as several studies and national registries have highlighted the heightened incidence of AF among those with worsening renal function. The European PREFER register in AF indicates that using formulas such as MDRD and CKD-EPI would assign patients to different dosages than those indicated by the CG formula [84, 85]. In patients with mild-to-moderate CKD (creatinine clearance (CrCl) 30–49 mL/min), the safety and efficacy of DOACs vs. warfarin was consistent with patients without CKD in landmark DOAC trials, hence the same considerations for stroke risk assessment and choice of oral anticoagulant (OAC) may apply. Moreover, observational studies showed that DOACs may be associated with lower risks of adverse renal outcomes than warfarin, conferring some grade of protection against the progression of renal failure [86, 87]. The CONFIRM-AF database demonstrates the superiority of DOACs over warfarin in patients with renal dysfunction [88]. In patients with CrCl 15–29 mL/min, RCT-derived data on the effect of VKA or DOACs are lacking. These patients were essentially excluded from the major RCTs, with the exception of apixaban, which was tested in 269 patients with ClCr between 25–30 mL/h in the ARISTOTLE trial [89]. The evidence for the benefits of OAC in patients with end-stage kidney disease with CrCl ≤15 mL/min or on dialysis is even more limited, and to some extent, controversial. Some studies compared apixaban versus warfarin in patients undergoing hemodialysis: the bleeding rate was not higher in the case of apixaban. More studies will be needed to confirm these initial data. While there is notable diversity in renal clearance, significant variability among different anticoagulant drugs is not observed. Dose reduction without specific criteria increases the risk of stroke and death, emphasizing the importance of proper dosing (Table 7) [90]. Future perspectives in anticoagulation therapy involve exploring novel therapeutic targets, such as factor XI, which promotes thrombus propagation by supporting thrombin production. Inhibitors of factor XI, such as milvexian and asundexian are being investigated in phase III studies to evaluate their efficacy and safety profile [91].

**Table 6.** **Mechanism of action and pharmacological characteristics of DOACs**.

	Dabigatran	Rivaroxaban	Apixaban	Edoxaban
Mechanism	Oral direct reversible competitive thrombin antagonist	Oral direct reversible competitive factor Xa antagonist	Oral direct reversible competitive factor Xa antagonist	Oral direct reversible competitive factor Xa antagonist
CYP3A4 substrate	No	Yes	Yes	Yes
Metabolism	Glucoronic acid conjugation	CYP3A4, CYP2J2	CYP3A4/5, CYP1A2, CYP2C8, CYP2C9, CYP2C19	CYP3A4/5
Bioavability	3%–7%	66% without food, 80%–100% with food	50%	62%
Prodrug	Yes	No	No	No
Absorption with food	No effect	39%	No effect	6%–22%
Clearance non renal/renal	20%–80%	35%–65%	73%–27%	50%–50%

CYP, cytochromes P450; Factor Xa, activated coagulation factor X; DOACs, direct oral anticoagulants.

**Table 7.** **eGFR-adjusted dosages for DOACs and other dose reduction criteria**.

eGFR category	Dabigatran	Rivaroxaban	Apixaban	Edoxaban
>95 mL/min	150 mg twice a day	20 mg once daily	5 mg twice daily	60 mg once daily
50–94 mL/min	150 mg twice a day	20 mg once daily	5 mg twice daily	60 mg once daily
30–49 mL/min	110 mg twice a day	15 mg once daily	5 mg twice daily	30 mg once daily
15–29 mL/min	Do not use	15 mg once daily	2.5 mg twice daily	30 mg once daily
Dialysis	Do not use	Do not use	Do not use	Do not use
Other dose reduction criteria:	Dabigatran	Rivaroxaban	Apixaban	Edoxaban
	Age >80 years old;	Not recommended if concomitant use of:	At least 2 of the following:	At least 2 of the following:
	Concomitant Verapamil treatment;	*CYP3A4* inhibitors and/or inducers;	Age >80 years old;	CrCl 15–50 mL/min;
	Consider dose reduction according to RBR if: high bleeding risk pathology [e.g., GERD, esophagitis, gastritis, etc.]	P Glycoprotein inhibitors;	Body weight <60 kg;	Body Weight <60 kg;
		Systemic azole antifungal drugs	Serum Cr levels >1.5 mg/dL	Concomitant use of Dronedarone, Erythromicin, Ciclosporin or Ketoconazole

eGFR, estimated glomerular filtration rate; mL/min, milliliter/minute; RBR, risk-to-benefit-ratio; GERD, gastroesophageal reflux disease; CYP, cytochromes P450; CrCl, creatinine clearance; DOACs, direct oral anticoagulants.

## 6. Venous Thromboembolism and Pulmonary Embolism 

Venous thromboembolism (VTE), clinically manifesting as deep vein thrombosis (DVT) or pulmonary embolism (PE), represents a prevalent disorder associated with significant morbidity and mortality. Globally it ranks as the third most frequent acute cardiovascular syndrome, following MI and stroke. Anticoagulation is the cornerstone of VTE treatment [92, 93].

### 6.1 Main Therapeutic Options

Historically, VKAs, UFH, or LMWH, were the primary choices for VTE treatment and prevention. Although effective, these agents pose significant drawbacks, including individual pharmacokinetics and pharmacodynamics, the necessity for subcutaneous and/or intravenous delivery, susceptibility to drug interactions and vitamin K intake for VKAs. Dietary habits, variations in alcohol consumption, and long-term changes often lead to significant fluctuations in INR values. DOACs offer several advantages, for example the oral administration at a fixed dose and the absence of mandatory routine laboratory monitoring, exerting direct action on coagulation factors with a predictable pharmacokinetic profile. However, DOACs exhibit extended elimination half-lives when compared to UFH or LMWH. Consequently, DOACs can accumulate in patients with suboptimal kidney function or impaired liver function. Pivotal RCTs for acute or extended VTE treatment excluded patients with serum creatinine levels >2.5 mg/dL or ClCr <25 to 30 mL/min [82]. Evidence-based clinical practice guidelines strongly advocate for the use of DOACs as the preferred choice for most patients with non-cancer-related VTE cases [92]. Notably, DOACs are not recommended for patients with severe kidney impairment, during pregnancy and lactation, and for individuals with antiphospholipid antibody syndrome or mechanical heart valves [82, 92, 94]. Failure to promptly initiate anticoagulation therapy and delayed administration of therapeutic anticoagulation therapy can result in a worse prognosis [94]. Therefore, it is imperative to utilize anticoagulants with rapid onset of action and a predictable dose-effect response for the acute treatment of VTE.

### 6.2 Treatment Regimens for DOACs

DOACs fulfill these criteria, offering a favorable pharmacodynamic profile and a dose-response curve. Two distinct treatment regimens for DOACs in VTE have been developed. The singular drug approach entails an initial phase of high-dose DOAC treatment, followed by a maintenance dose without parenteral anticoagulants. Conversely, the sequential approach comprises an initial treatment with heparin or fondaparinux for 5 to 10 days, followed by a maintenance dose of DOACs. Apixaban and rivaroxaban have been developed using the single drug approach, while dabigatran and edoxaban using the sequential approach (Table 8, Ref. [95, 96, 97, 98, 99, 100, ]). Large-scale Phase III global RCTs have systematically assessed fixed doses of DOACs in comparison to conventional anticoagulation therapy (heparin followed by VKA) for VTE treatment. These trials demonstrated the non-inferiority of each DOAC concerning efficacy compared to conventional treatment [95, 96, 97, 98, 101, 102]. Furthermore, a meta-analysis of these clinical trials corroborated the non-inferiority of DOACs in efficacy, coupled with a noteworthy reduction in bleeding risk [103]. Despite the decline in warfarin use, it remains a viable treatment option for patients with severe renal insufficiency, antiphospholipid syndrome, or financial constraints hindering DOAC accessibility. There are uncertainties surrounding the tradeoffs associated with the use of DOACs based on certain indications [82]. For example, in the context of catheter-associated DVT, Brandt *et al*. [104] found that apixaban 2.5 mg twice daily, in comparison to a placebo, was linked to decreased rates of VTE with no significant difference in major bleeding. In the TRIM-line study, thromboprophylaxis with rivaroxaban 10 mg daily, compared to placebo, showed no significant variation in the rate of VTE among patients with cancer and central venous catheters (CVCs), except for one major bleeding incident in the rivaroxaban group [105]. An ongoing trial, aiming to enrol 1828 patients, is presently comparing rivaroxaban 10 mg daily with a placebo for primary thromboprophylaxis in cancer patients with CVCs. Regarding cerebral venous sinus thrombosis, findings from the RE-SPECT CVT indicate that dabigatran 150 mg twice daily, when compared to warfarin with an INR of 2 to 3, led to no recurrent VTE in both groups [106]. One major bleeding event was recorded in the dabigatran arm, and two in the warfarin arm at 25 weeks. Concerning splanchnic vein thrombosis, the RIPORT study revealed that rivaroxaban 15 mg daily, compared to placebo, resulted in a significantly lower rate of recurrent VTE in patients with noncirrhotic chronic portal vein thrombosis [107]. Nevertheless, the sample size is limited, so it is essential to investigate the rates of recurrent VTE, and additional RCTs are needed.

**Table 8.** **DOAC for VTE with associated RCTs**.

RCTs	DOAC	Mechanism of action	Dose and Regimen
AMPLIFY [97]	Apixaban	Factor Xa inhibitor	10 mg twice daily for 7 days, then 5 mg twice daily
RECOVER [98]	Dabigatran	Direct thrombin inhibitor	150 mg twice daily after 5–10 days of parenteral anticoagulation
RECOVER-II [99]			
HOKUSAI-VTE [100]	Edoxaban	Factor Xa inhibitor	60 mg once daily after 5–10 days of parenteral anticoagulation (reduce dose to 30 mg daily for CrCl ≤50 mL/min, body weight ≤60 kg or in patients taking P-glycoprotein inhibitors)
EINSTEIN-DVT [95]	Rivaroxaban	Factor Xa inhibitor	15 mg twice daily for 21 days, then 20 mg once daily
EINSTEIN-PE [96]			

VTE, venous thromboembolism; DOAC, direct oral anticoagulant; RCTs, randomized controlled trials; CrCl, creatinine clearance.

### 6.3 Treatment Phases

Treatment phases for deep vein thrombosis (DVT) are categorized into three distinct stages (Table 9):

- Initial treatment (5–21 days after diagnosis): patient management involves parenteral therapy transitioning to VKA or DOACs administered at high doses.

- Long-term treatment (3–6 months): both VKA and DOACs are used during this phase. These stages, initial and long-term, are mandatory for all DVT patients [108].

- Extended phase: continuation of treatment beyond the initial 3–6 months depends on evaluating the RBR of prolonged anticoagulation [109].

**Table 9.** **VTE treatment phases**.

Antithrombotic treatment	Initial treatment (days 5 to 21)	Long-term treatment (3 to 6 months)	Extended treatment (After 3 or 6 months)
Apixaban	10 mg twice a day for 7 days	5 mg twice a day	2.5 mg twice a day after 6 months
Rivaroxaban	15 mg twice a day for 21 days	20 mg once daily	10 mg or 20 mg once daily beyond 6 months
Edoxaban	60 mg once daily (30 mg if ClCr <50–30 mL/min or concomitant potent PP-inhibitors) preceded by LMWH for 5 to 10 days		
Dabigatran	150 mg preceded by LMWH for 5 to 10 days		
VKA	Achieve INR 2–3 preceded by LMWH for 5 to 10 days		

LMWH, low molecular weight heparin; INR, international normalized ratio; VTE, venous thromboembolism; VKA, vitamin K antagonists; CrCl, creatinine clearance.

After PE diagnosis, the optimal duration of treatment has been explored in four randomized clinical trials focusing on dabigatran [99], rivaroxaban [110], apixaban [111], and edoxaban [102]. These studies confirmed the efficacy of these medications in reducing recurrence risks but with an increased bleeding risk. Each study had a condition of clinical equipoise about continuing oral anticoagulant therapy, prescribing up to 12 months of initial DOAC or warfarin treatment post-VTE. Afterwards, patients were assigned to active treatment or placebo, except for two studies that included an active comparator in the control group [99, 110]. Key findings are summarized in **Supplementary Table 3**. Notably, the RE-MEDY study found that 150 mg of dabigatran given twice daily was associated with a higher recurrence risk compared to warfarin [99]. Extended anticoagulant therapy beyond the typical 3-month period hinges on the estimated risk of recurrence after stopping treatment. The PADIS-PE trial found that warfarin for an additional 18 months following an initial 6 months after PE reduced recurrent VTE and major bleeding risks [112]. While prior studies focused on extended DOAC treatment for VTE including DVT, none specifically addressed extended DOAC treatment post-PE. An observational study indicated that extending anticoagulation for PE for 2–12 years was more beneficial and safer than not extending treatment [113]. Still, indefinite OAC therapy must be carefully weighed against bleeding risk [114]. Although results generally support extended treatment, pinpointing which patients will benefit most remains challenging. Few studies have examined extended treatment in VTE [115]. DeRemer *et al*. [116] analyzed the impact of continued treatment using 2.5 mg versus 5 mg apixaban in patients 6 months-post VTE treatment, suggesting comparable outcomes with the lower dose. However, this study is limited by potential biases due to its observational design. Chopard *et al*. [113] conducted a cohort study of 1199 patients post-PE. They found that 71.5% underwent extended treatment with DOACs or VKA for at least two years. Extended treatment was associated with a 2.1% risk of all-cause death or recurrent VTE, versus a 7.7% risk without extended treatment [113]. Determining anticoagulation duration should involve individual patient risk factor analysis. Kyrle *et al*. [117] assessed the Vienna Prediction Model (VPM) to estimate recurrence probability in unprovoked VTE by considering sex, thrombosis site, and D-dimer levels. During a median follow-up of 23.9 months, the study found a 5.2% one-year recurrence rate. While the VPM validation aids in optimizing extended-phase anticoagulation in unprovoked VTE, further research is needed to reliably estimate bleeding complications and enhance decision-making, especially now that reduced dose DOACs have become more prevalent than VKA for extended-phase treatment [118].

### 6.4 Antithrombotic Prophylaxis for Cancer-Associated Thromboembolism

Patients in certain special categories, such as those with cancer or thrombophilia, face a heightened risk of developing VTE, leading to complex clinical management. Outpatient oncology patients exhibit nearly a 5-fold higher probability of VTE development compared to non-cancer patients, with a corresponding 2 to 3-fold higher mortality rate [119]. Its management in cancer patients with solid tumors is complex due to an increased risk of both thrombotic and hemorrhagic events [120]. Current guidelines from the American Society of Hematology (ASH) recommend using either LMWH or a DOAC, specifically apixaban 2.5 mg or rivaroxaban 10 mg, for VTE prevention in high-risk patients [121]. Pharmacological prophylaxis is emphasized in high-risk patients to prevent thrombotic complications. The AVERT trial examined the incidence of VTE in two groups of outpatient cancer patients at medium-high risk, randomized to receive apixaban 2.5 mg twice daily or placebo. VTE occurred in 4.2% in the apixaban group and 10.2% in the placebo group (hazard ratio, 0.41; 95% confidence interval [CI], 0.26 to 0.65; *p* < 0.001). The rate of major bleeding episodes was higher with apixaban than with placebo [122]. A meta-analysis highlighted the effectiveness of anticoagulant prophylaxis in reducing VTE incidence in outpatient cancer patients. Both oral and parenteral anticoagulants have been studied, with apixaban demonstrating a reduction in the risk of VTE without increasing bleeding risk [123]. However, validation in larger study cohorts is warranted. The effectiveness of LMWH-based thromboprophylaxis has been supported by various randomized trials, including the PROTECHT and SAVE-ONCO studies, further confirming its net benefit [124, 125]. The guidelines typically recommend a minimum of 6 months of anticoagulation treatment after cancer-associated VTE [126]. While LMWH remains the preferred anticoagulant drug class for patients with GI and urogenital tract cancer, DOACs are recommended as first-line treatment in almost all other patients with cancer-associated thrombosis (clinical trials comparing DOAC versus LMWH for cancer-associated VTE are summarized in **Supplementary Table 4**). However, there are uncertainties regarding their treatment beyond the initial 6-month period [127]. Despite the guidelines suggesting continued anticoagulation therapy beyond the initial 6-month period, specific dosage recommendations or decision tools are lacking, with uncertainties persisting. Studies like SELECT-D: a 12 month study and the Cancer-DACUS trial indicate lower VTE recurrence with continued treatment, but a higher risk of bleeding compared with the placebo group [128, 129]. Rivaroxaban, apixaban, and LMWH are potential options for primary prevention in high-risk oncology patients in the absence of significant contraindications, as per ESC guidelines, particularly those with locally advanced or metastatic lung or pancreatic cancer and a Khorana score >2 [130].

### 6.5 Antithrombotic VTE Prophylaxis for Patients with Thrombophilia

Patients affected by inherited thrombophilia (IT) face a heightened risk of DVT complicated by PE or thrombosis in atypical sites at a young age. In fact, a thrombophilic phenotype occurs in approximately 4% of patients with idiopathic VTE [131]. Genetic testing is clinically useful in carriers of severe IT, notably those with confirmed deficiency of antithrombin, protein C, or protein S, and those with homozygous Factor V Leiden, homozygous prothrombin variant G20210A, or heterozygous for the two combined genetic abnormalities. These patients are candidates for indefinite anticoagulant treatment after the first episode of VTE. In VTE trials with DOACs, the prevalence of known thrombophilia ranges from 2 to 18% [99]. Key studies, such as RE-COVER, RE-COVER II and RE-MEDY comparing dabigatran with warfarin [98], EINSTEIN studies comparing rivaroxaban with warfarin [99, 110], and AMPLIFY and HOKUSAI studies comparing warfarin with apixaban and edoxaban, respectively, have been conducted [111, 132]. A Post-hoc analysis of these studies reveals no discernible differences in the efficacy and safety of DOACs, regardless of the presence or absence of IT [131].

## 7. Cardiac Surgery and Structural Interventional Procedures

Structural interventions and cardiac surgery pose an increased risk of thrombotic and haemorrhagic events. The thrombotic risk peaks in the initial months, and is even more pronounced after tricuspid valve interventions due to the low pressures in the right sections of the heart. Guidelines suggest that if a patient already has an indication for DOAC or VKA therapy, it is recommended to continue the therapy unless there is a high risk of bleeding. **Supplementary Tables 5,6** provide a summary of the most relevant evidence from the literature supporting antithrombotic treatment after structural interventions and cardiac surgery.

### 7.1 Coronary Artery Bypass Graft Surgery

The goal of ATT in coronary artery bypass graft (CABG) surgery patients would be to reduce disease progression and graft occlusion, although there is no evidence of this and data are scarce. Compared with aspirin monotherapy, the benefit of DAPT following CABG is still controversial. Current guidelines recommend a 12-month duration of DAPT in ACS patients, including those undergoing CABG [133] and new data are emerging for the possibility of using P2Y12 inhibitors as a second antiplatelet drug in combination with aspirin [134]. However, the optimal duration of DAPT in CABG patients with CCS and the potential role of DOACs as a therapeutic option remain uncertain. In individuals with both CABG and concurrent AF, guidelines suggest using a single DOAC alongside antiplatelet therapy (aspirin or clopidogrel), despite the lack of randomized trials assessing its efficacy and safety. Similarly, the combination of DAPT with a DOAC appears to pose a heightened risk of haemorrhage, although robust evidence is scarce. Following CABG, single therapy may be discontinued after one year, with DOAC as a single treatment option. The CASCADE study, involving 113 patients randomized to either DAPT or aspirin monotherapy, showed no improvement in venous bypass patency in the DAPT group [135]. A sub-analysis of the CURE study, with 2072 patients undergoing CABG, found that those taking DAPT had a reduction in the incidence of cardiovascular death, MI, and stroke at one year [136]. However, there was a 30% increase in life-threatening haemorrhage. In the DACAB study, greater patency of venous bypasses was observed in patients on DAPT compared to aspirin alone, with no difference between ticagrelor and aspirin treatment. The study also revealed an increase in minor bleeding in the DAPT group [137].

### 7.2 Transcatheter Aortic Valve Implantation

Transcatheter aortic valve implantation (TAVI) is employed in patients with symptomatic severe aortic stenosis, typically aged over 75 years, and who are not suitable candidates for surgical aortic valve replacement. Corrective intervention is recommended in symptomatic patients with high-gradient aortic stenosis (Vmax >4 m/s, mean gradient (MG) >40 mmHg, valve area <1 ${\mathrm{cm}}^{2}$), in patients with low flow-low gradient aortic stenosis with reduced ejection fraction presenting with contractile reserve. Surgery is recommended in asymptomatic patients with left ventricular dysfunction not attributable to other causes or in patients with documented symptoms during stress testing. The management of peri-procedural antithrombotic therapy depends on the administered molecule and the concurrent clinical characteristics of the patient. Patients receiving VKA therapy should undergo discontinuation to achieve a target INR <1.5. However, in cases of mechanical heart valves, atrial fibrillation with significant mitral stenosis, or thrombotic events within the previous four weeks, bridging with an oral anticoagulant (OAC) is recommended using UFH or therapeutic doses of LMWH [138]. Also, the guidelines recommend lifelong single antiplatelet therapy (SAPT) after the procedure in patients for whom an OAC is not indicated. However, OAC is advised indefinitely if there is another indication for such therapy [139]. The Delphi Consensus recommends an optimal duration of single antithrombotic therapy ranging from 3 to 12 months, based on individual risk [138]. Conversely, the ARTE trial compared clopidogrel plus aspirin with aspirin alone in patients undergoing TAVI, revealing that dual antiplatelet administration increased bleeding risk without reducing mortality, stroke and MI three months post-procedure [140]. The POPular-TAVI trial represents a pioneering RCT examining the efficacy of anticoagulation alone versus anticoagulation combined with antiplatelet therapy in patients undergoing TAVI [141]. Two cohorts were examined: one, without DOAC indication, and the second with indication for oral anticoagulation, randomized in a 1:1 ratio to receive clopidogrel or not. Initial findings revealed a lower incidence of severe bleeding with anticoagulant therapy alone compared to the cohort taking DOAC combined with clopidogrel, without a reduction in thromboembolic events. Limitations of this study include: small sample size and limited use of DOACs [142]. The GALILEO trial investigated patients undergoing successful TAVI without indication for DOAC therapy [141]. Patients were randomized to receive rivaroxaban 10 mg daily plus aspirin versus antiplatelet therapy alone. The primary outcome, a composite of mortality from thromboembolic causes and events, revealed higher rates in the rivaroxaban group over approximately 18 months of follow-up, alongside increased severe bleeding [143]. In contrast, the ATLANTIS trial compared antiplatelet therapy to apixaban in TAVI patients [144]. Although not superior to standard therapy in primary outcomes, apixaban showed a lower thrombotic risk. Notably, patients without anticoagulant indication outside TAVI had increased non-cardiovascular mortality with apixaban, warranting further investigation [144]. Two ongoing trials, ACASA-TAVI [145] and AVATAR-TAVI [146], aim to further explore the optimal antithrombotic strategy post-transcatheter aortic valve replacement (TAVR), with ACASA-TAVI focusing on the use of OACs vs. single antiplatelet therapy, and AVATAR-TAVI investigating single anticoagulant superiority over the combination with aspirin. Table 10 (Ref. [140, 141, 143, 145, 147]) provides a summary of the relevant completed or ongoing trials on antithrombotic treatments for TAVR.

**Table 10.** **Completed or ongoing trials on antithrombotic treatments for TAVR**.

Comparison of antithrombotic treatment strategies	SAPT	DAPT	OAC+SAPT
OAC	ACASA-TAVI [145]	ADAPT-TAVR [147]	POPULAR TAVI B [141]
OAC+SAPT	-	GALILEO [143]	-
DAPT	ARTE [140]	-	-
	POPular TAVI [141]		

SAPT, single antiplatelet agent; DAPT, dual antiplatelet therapy; OAC, oral anticoagulant; TAVI, transcatheter aortic valve implantation; TAVR, transcatheter aortic valve replacement.

### 7.3 Mitral valve Transcatheter Edge-to-Edge-Repair

Mitral Valve Transcatheter Edge-to-Edge-Repair (M-TEER) serves as a nonsurgical alternative for patients experiencing signs of severe mitral valve (MV) regurgitation, despite optimized medical therapy and adherence to specific echocardiographic criteria [148]. Notably, the COAPT trial involved patients without a pre-existing indication for DOAC therapy, who received aspirin and/or clopidogrel for at least 6 months after M-TEER [149]. In the MITRA-FR trial, 78 patients were administered DAPT with aspirin and clopidogrel for 3 months, followed by aspirin monotherapy [150]. In the EVEREST II trial, 77 patients received DAPT with aspirin and clopidogrel for 30 days, followed by aspirin monotherapy for 6 months [151]. However, available studies are limited, data are scarce, and standardized protocols are lacking. More robust data are eagerly awaited to confirm the true benefit of antithrombotic therapy in this context, even though aspirin treatment seems efficient in reducing the risk of death and thromboembolism [152].

### 7.4 Left Atrial Appendage Occlusion

Transcatheter left atrial appendage occlusion (LAAO) is a valuable alternative option for stroke and systemic embolism in patients unsuitable for OACs due to HBR or contraindications. ATT after LAAO is essential to prevent thrombus formation on the device, reducing embolic risk [153, 154]. PROTECT-AF and PREVAIL trials recommend a short-term VKA plus SAPT, followed by long-term SAPT, soon after WATCHMAN (Boston Scientific, Natick, MA, USA) implantation [155, 156]. Limited data exist on DOAC regimen after LAAO. The ADRIFT trial found no differences in adverse events between rivaroxaban and DAPT groups [157]. The continuation of SAPT after 6 month-long dual therapy is debated, due to bleeding risk, but aspirin monotherapy is common. In general, continuing aspirin appears reasonable if a concomitant indication for long-term antiplatelet therapy coexists, but its benefit-risk profile has to be carefully discussed with the patient otherwise. To date, the optimal antithrombotic therapy in patients undergoing transcatheter LAAO remains debated [153].

### 7.5 Patent Foramen Ovale

Transcatheter device-based closure of patent foramen ovale (PFO) is advised for patients under 60 with cryptogenic stroke and high-risk characteristics, such as an atrial septal aneurysm or a moderate-to-severe right-to-left shunt. Typically, SAPT is administered during the pre-intervention phase [158, 159]. The PFO CLOSE trial randomized patients to either transcatheter closure or medical management using anticoagulants or antiplatelet drugs [160]. This trial reported a reduced rate of stroke recurrence at five years with anticoagulation; however, the significance of these findings was not assessed due to the study’s limited scope on clinical outcomes. The ongoing HALTI study (NCT04475510) is set to evaluate the benefits of discontinuing antiplatelet therapy 12 months following a successful PFO closure. Current guidelines from Professional Cardiology Societies suggest using DAPT (aspirin plus clopidogrel) for 1 to 6 months post-closure, transitioning to SAPT, preferably with aspirin, for the following 5 years. Patients with additional cardiovascular risks or a high potential for recurrent cerebrovascular events might consider extended ATT beyond this period. Alternatively, for patients at HBR or those with a low predicted risk of stroke recurrence, halting all antithrombotic treatments a year post-procedure is considered a viable option [161].

## 8. Antithrombotic Treatments for Pregnant Women

### 8.1 Coronary Artery Disease

The management of ATT during pregnancy poses a significant challenge for cardiologists and multidisciplinary teams. ACS is increasingly becoming a notable complication of pregnancy, given the rising mean age of pregnancy [162]. Furthermore, ACS is not solely of atherosclerotic origin: there is a notable incidence of spontaneous acute coronary dissections and coronary thrombosis due to hypercoagulability [163, 164]. The management of ACS during pregnancy largely follows established guidelines outside of pregnancy. However, concerning medication use, evidence is limited. While low-dose aspirin was proven to be relatively safety, there was a lack of reliable data for bivalirudin, prasugrel, and ticagrelor [165]. Clopidogrel is currently the only P2Y12 inhibitor with more substantial data [166]. Following a myocardial infarction with coronary stent placement, it is advisable to wait at least 12 months before planning a pregnancy. This timeframe aims to avoid premature cessation of DAPT, thereby preserving coronary stability [165].

### 8.2 Pre-Eclampsia

The use of an antiplatelet drug such as aspirin has been linked to the prevention of pre-eclampsia, a significant cause of maternal and foetal morbidity and mortality. Numerous studies and meta-analyses conducted in recent decades have identified antiplatelet agents as effective in reducing the risk of pre-eclampsia [167, 168, 169]. The timing of antiplatelet therapy initiation has been largely investigated, with some meta-analyses suggesting no significant influence on pre-eclampsia prevention outcomes [170], while others emphasize the need for early initiation within the first 16 weeks [168, 171, 172]. Contrarily, the ASPRE trial randomized pregnant women at high risk of pre-eclampsia to receive low-dose aspirin (150 mg) versus placebo, achieving a statistically significant lower incidence [173]. The ASPIRIN study investigated the use of low-dose aspirin (81 mg) in nulliparous women, resulting in a reduction in the incidence of preterm birth before 37 weeks and a decrease in perinatal mortality [174]. The EAGeR trial involved women with a history of recurrent pregnancy loss (recurrent spontaneous abortions), where the use of low-dose aspirin led to a reduced risk of foetal loss, an increase in live births, and a decreased risk of preterm birth compared to placebo [175]. The use of aspirin for the prevention of preeclampsia in high-risk women is recommended, but adequate monitoring and individual assessment of risks and benefits are essential.

### 8.3 Venous Thromboembolism

VTE exhibits a higher incidence during pregnancy and the postpartum period, compared to the general population [176, 177], with the highest risk in the immediate post-delivery period [178, 179]. Prophylactic treatment for VTE predominantly relies on the use of LMWH, established as the drug of choice [180]. Optimal thromboprophylaxis is achieved when the LMWH dosage is tailored to body weight at the initial prenatal visit [181]. LMWH remains the drug of choice for the treatment of hemodynamically stable PE and DVT. UFH is employed in the treatment of massive PE cases with a risk of hemodynamic instability, albeit with an increased risk of thrombocytopenia. Thrombolytic agents should be reserved for patients experiencing severe hypotension or shock, acknowledging the non-negligible risk of bleeding. Fondaparinux emerges as a viable alternative in cases of allergy or adverse reactions to LMWH. DOACs are currently not indicated for use in pregnant patients [165]. Specifically, rivaroxaban crosses the placental barrier and is therefore not recommended during pregnancy, as it may be associated with a risk of spontaneous abortion and possible embryopathy [182].

### 8.4 Atrial Fibrillation

DOACs are contraindicated during pregnancy, even for AF management. There are two alternative options: therapeutic doses of LMWH throughout the entire duration of pregnancy, spanning all three trimesters, or LMWH during the first and third trimesters with VKA during the second one [165].

### 8.5 Mechanical Heart Valves

Unfortunately, pregnancy and the presence of mechanical valves are associated with a high rate of maternal-foetal complications, stemming from the delicate balance between appropriate anticoagulation and the risks of bleeding and prosthesis thrombosis [183]. While UFH and LMWH are linked to a high risk of prosthetic heart valve thrombosis [183, 184], the risk is relatively low with VKAs during pregnancy (0–4%) [184, 185, 186]. The LMWH dose requirement significantly increases due to elevated renal clearance, but monitoring anti-Xa levels with dose adjustments reduces the risk. Nevertheless, the safety of LMWH for thrombosis risk remains debated, regardless of the trimester of use. Currently, the use of VKAs with closely monitored INR is the safest regimen to prevent valve thrombosis. VKAs during the first trimester are associated with an increased risk of spontaneous abortion, linked to escalating doses [185, 186]. Additionally, VKAs in the first trimester may cause embryopathy, particularly limb defects and nasal hypoplasia, while their use in the second and third trimesters may be associated with ocular and central nervous system anomalies and intracranial haemorrhage [183]. Vaginal delivery is not recommended if VKAs are used in the last trimester due to the risk of foetal intracranial haemorrhage. UFH and LMWH, on the other hand, do not cross the placenta. The advantages and disadvantages of different anticoagulation regimens should be extensively discussed before pregnancy. VKAs are more effective in preventing valve thrombosis, ensuring greater safety for the mother, but the risks of embryopathy or fetopathy, foetal loss, and foetal bleeding are not negligible. On the other hand, with LMWH, there are fewer foetal risks but a higher risk of valve thrombosis. The management of valvular thrombosis should be approached similarly to the non-pregnant state [165]. Choosing the correct anticoagulation regimen is a genuine challenge, requiring thorough discussion with the mother, who should be informed about the RBR for herself and the foetus, and should be an integral part of the decision-making process.

## 9. Conclusions with a Focus on Future Perspectives

ATT is widely employed in the management of cardiovascular diseases and constitutes a field of research in constant and fervent renewal. The growing evidence supporting shorter-duration therapies in ACS [187], novel coagulation factors [e.g., FXIa] to be targeted in anticoagulant therapies [91], or the growing evidence on the integration of genetic characterization in risk score formulation [188], are only some of the examples that cannot be overlooked. Therefore, an in-depth and up-to-date understanding of its indications, along with a prospective outlook on potential future implications, is fundamental for clinically tailored management which is aligned to the individual needs of each patient.

## Abbreviations

ACS, acute coronary syndrome; AF, atrial fibrillation; ASH, American Society of Hematology; ATT, antithrombotic treatments; CABG, coronary artery bypass graft; CAD, coronary artery disease; CCS, chronic coronary syndrome; CKD, chronic kidney disease; CVC, central venous catheter; CVD, cardiovascular diseases; DAPT, dual antiplatelet therapy; DES, drug eluting stent; DOAC, direct oral anticoagulant; DVT, deep vein thrombosis; ESC, European Society of Cardiology; GI, gastrointestinal; HBR, high bleeding risk; INR, international normalized ratio; IT, inherited thrombophilia; LAAO, left atrial appendage occlusion; LMWH, low molecular weight heparin; MACE, major adverse cardiovascular events; MI, myocardial infarction; M-TEER, mitral valve transcatheter edge-to-edge-repair; MV, mitral valve; NACE, net adverse cardiovasculare events; NSTEMI, non ST segment elevation MI; PAD, peripheral artery disease; PCI, percutaneous coronary intervention; PE, pulmonary embolism; PFO, patent foramen ovale; RBR, risk-benefit-ratio; RCT, randomized control trial; SAPT, single antiplatelet therapy; STEMI, ST segment elevation MI; TAT, triple antithrombotic therapy; TAVI, transcatheter aortic valve implantation; TIA, transient ischemic attack; TMVR, transcathether mitral valve replacement; TTR, time in therapeutic range; TTVR, transcatheter tricuspid valve replacement; UFH, unfractioned heparin; VHD, valvular heart disease; VKA, vitamin k antagonist; VPM, Vienna Prediction Model; VTE, venous thromboembolism.

## Supplementary Material

Supplementary material associated with this article can be found, in the online version, at https://doi.org/10.31083/j.rcm2508281.
