# Supplementary material for: Current Antithrombotic Treatments for Cardiovascular Diseases: A Comprehensive Review
Source: Rev Cardiovasc Med. 2024 Aug 8;25(8):281. doi: 10.31083/j.rcm2508281 (PMC11366999; doi:10.31083/j.rcm2508281)
Supplement: Supplementary file 1 [file 2153-8174-25-8-281-s1.docx]

**Supplementary Material:**

**Current Antithrombotic Treatments for
Cardiovascular Diseases: a comprehensive review**

**Supplementary Table 1: Characteristics of studies on antithrombotic treatments for Chronic Coronary Syndrome.**

| **Clinical Trial** | **Randomisation arms** | **Follow-up** | **No. Patients** | **Main Efficacy end points** | **Main safety end points/results** |
| --- | --- | --- | --- | --- | --- |
| [**CHARISMA**](https://www.nejm.org/doi/full/10.1056/nejmoa060989) | A: daily clopidogrel 75 mg + aspirin 75–162 mg  B: daily placebo + aspirin 75–162 mg | median 28 months | 15,603 | Composite end point of CV death, MI, or stroke: RR 0.93, 95% CI 0.83–1.05, p = 0.22 | Severe bleeding ([GUSTO](https://www.nejm.org/doi/full/10.1056/NEJM199309023291001)): 1.71% vs 1.3, p = 0.09 |
| [**PRODIGY**](https://www.ahajournals.org/doi/10.1161/CIRCULATIONAHA.111.071589) | A: daily clopidogrel 75 mg + aspirin 80-160 mg for 24 months  B: daily clopidogrel 75 mg + aspirin 80-160 mg for 6 months | 2 years | 2013 | Composite of death of any cause, myocardial infarction, or cerebrovascular accident: HR 0.98, 95 % CI 0,74-1,29, p=0,91 | Composite of type 5, 3 and 2 bleeding ([BARC](https://www.ahajournals.org/doi/10.1161/CIRCULATIONAHA.110.009449)): HR 0,46, 95 % CI 0.31 - 0.69, p = 0,00018  Rate of bleeding ([TIMI](https://www.sciencedirect.com/science/article/pii/0735109788901581?via%3Dihub)): HR 0.55, 95 % CI 0.3-1.04, p=0.63 |
| [**EXCELLENT**](https://www.ahajournals.org/doi/10.1161/CIRCULATIONAHA.111.059022?url_ver=Z39.88-2003&rfr_id=ori:rid:crossref.org&rfr_dat=cr_pub%20%200pubmed) | A: daily clopidogrel 75 mg + aspirin 100-200 mg for 6 months  B: daily clopidogrel 75 mg + aspirin 100-200 mg for 12 months | 12 months | 1143 | Target vessel failure defined as a composite of cardiac death, myocardial infarction, or ischemia-driven target vessel revascularization at 12 months: HR 1.14, 95 % CI 0.7-1.86, p=0.60 | Composite of death, myocardial infarction, stroke, ST, or TIMI major bleeding: HR 1.15, 95% CI 0.64-2.06, p= 0.64 |
| [**ISAR-SAFE**](https://academic.oup.com/eurheartj/article/36/20/1252/2293230) | A: daily clopidogrel 75 mg + aspirin 81-200 mg for 6 months  B: daily clopidogrel 75 mg + aspirin 81-200 mg for 12 months | 9 months | 4005 | Composite of death, myocardial infarction, ST (definite or probable), stroke, or TIMI major bleeding: HR 0.91, 95% CI 0.55–1.50, P for non inferiority < 0.001 | TIMI major bleeding: HR 0.8, 95 % CI 0.21-2.98, p=0.74  BARC bleeding: HR 0.49, 95 % CI 0.31-0.77, p=0.002 |
| [**CAPRIE**](https://www.thelancet.com/journals/lancet/article/PIIS0140-6736(96)09457-3/fulltext) | A: daily clopidogrel 75 mg  B: daily aspirin 325 mg | from 1 to 3 years (median 1,9 years) | 19,185 | Composite of ischaemic stroke, MI, or vascular death: RRR of 8.7%, 95% Cl 0.3–16.5, p = 0.043 | Rash (578 vs 442), diarrhoea (428 vs 332), upper GI discomfort (1441 vs 1686), intracranial bleeds (34 vs 47), GI bleeds (191 vs 255), neutropenia (10 vs 16) |
| [**EUCLID**](https://www.nejm.org/doi/full/10.1056/nejmoa1611688) | A: Ticagrelor 90 mg twice daily  B: Clopidogrel 75 mg once daily | 30 months | 13.885 | Composite of cardiovascular death, myocardial infarction, or ischemic stroke: HR 1.02, 95 % CI 0.92-1.13, p=0.65 | TIMI major bleeding: HR 1.10, 95 % CI 0.84-1.43, p=0.49 |
| [**DAPT**](https://www.nejm.org/doi/full/10.1056/nejmoa1409312) | A: daily aspirin 75–162 mg + clopidogrel 75 mg or prasugrel 10 mg  B: daily aspirin 75–162 mg + placebo | 30 months post PCI (18 months post randomisation) | 9961 | ST: 0.4% vs 1.4%, HR 0.29, 95% CI 0.17–0.48, p < 0.001  Major adverse CV and cerebrovascular events: 4.3% vs 5.9%, HR 0.71, 95% CI 0.59–0.85, p < 0.001 | Moderate or severe bleeds (GUSTO): 2.5% vs. 1.6%, p = 0.001 |
| [**PEGASUS TIMI 54**](https://www.nejm.org/doi/full/10.1056/nejmoa1500857) | A: Ticagrelor 90 mg twice daily + aspirin  A’: Ticagrelor 60 mg twice daily + aspirin  B: placebo + aspirin | Median 33 months | 21.162 | Composite of CV death, MI, stroke: A vs B: HR 0.8, 95 % CI 0,75-0,96, p = 0.008;  A’ vs B: 0.84, 95% CI 0.74-0.95, p = 0.004 | TIMI major bleeds: 2.6 % in A vs 2.3 % in A’ vs 1.06 % in B (p < 0.001 for A or A’ vs B) |
| [**COMPASS**](https://www.nejm.org/doi/full/10.1056/nejmoa1709118) | A: rivaroxaban 2.5 mg twice daily. + aspirin 100 mg  A’: rivaroxaban 5 mg twice daily  B: aspirin 100 mg | 23 months | 27.395 | Composite of CV death, MI or stroke: 4.1 % vs 4.9 % vs 5.4 % in A vs A’ vs B; p < 0.001 for A vs B; p = 0.12 for A’ vs B | Major bleeds (modified [ISTH](https://www.jthjournal.org/article/S1538-7836(22)15843-5/fulltext)) A vs B: 3.1 % vs 1.9 %, HR 1.70, 95 % CI, 1.4 -2.05, p < 0.001  Fatal bleeds A or A’ vs B: non significant  Intracranial bleeds A vs B: 0.3 % vs 0.3 %, p = 0.60 |
| [**PIONEER AF-PCI**](https://www.nejm.org/doi/full/10.1056/nejmoa1611594) | A: rivaroxaban 15 mg daily + a P2Y12 inhibitors  A’: rivaroxaban 2,5 twice daily + DAPT  B: VKA + DAPT | 12 months | 2124 | Composite secondary efficacy end point of death from cardiovascular cause, myocardial infarction, or stroke: HR 1.08, 95 % CI 0.69-1.68, p=0.75 for A vs B; HR 0.93, 95 % CI 0.59-1.48, p=0.76 for A’ vs B | Composite primary safety end point of TIMI major or minor bleeding or bleeding requiring medical attention: HR 0.59, 95 % CI 0.47-0.76, p < 0.001 for A vs B; HR 0.63, 95 % CI 0.50-0.80), p < 0.001 for A vs A’; HR 0.61, 95 % CI 0.50-0.75, p < 0.001 for A+A’ vs B |
| [**RE-DUAL PCI**](https://www.nejm.org/doi/full/10.1056/nejmoa1708454) | A: dabigatran 110 mg twice daily + a P2Y12 inhibitor  A’: dabigatran 150 mg twice daily + a P2Y12 inhibitor  B: VKA + DAPT | 14 months | 2725 | Composite secondary efficacy end point of thromboembolic events (myocardial infarction, stroke, or systemic embolism), death, or unplanned revascularization (PCI or coronary-artery bypass grafting): HR 1.13, 95 % CI 0.90-1.43, p=0.30 for A vs B; HR 0.89, 95 % CI 0.67-1.19, p=0.44 for A’ vs B; HR 1.04, 95 % CI 0.84-1.29, p=0.74 for A+A’ vs B | First major or clinically relevant nonmajor bleeding event as defined by ISTH (primary safety end point): HR 0.52, 95 % CI 0.42-0.63, p < 0.001 for A vs B; HR 0.72, 95 % CI 0.58-0.88, p=0.002 for A’ vs B |
| [**AUGUSTUS**](https://www.nejm.org/doi/full/10.1056/NEJMoa1817083) | A: apixaban 5 mg twice daily + DAPT  A’: apixaban 5 mg twice daily + a P2Y12 inhibitor  B: VKA + DAPT  B’: VKA + a P2Y12 inhibitor | 6 months | 4614 | Composite secondary efficacy outcome of all-cause death or ischemic event (including stroke, MI, ST definite/probable, or urgent revascularization): HR 0.93, 95 % CI 0.75-1.16, p NS for A+A’ vs B+B’; HR 0.89, 95 % CI 0.71-1.11, p NR for A+B vs A’+B’ | Major or CRNM ISTH (primary safety outcomes): HR 0.69, 95 % CI 0.58-0.81, p < 0.001 for A+A’ vs B+B’; HR 1.89, 95 % CI 1.59-2.24, p < 0.001 for A+B vs A’+B’ |
| [**ENTRUST-AF PCI**](https://www.thelancet.com/article/S0140-6736(19)31872-0/fulltext) | A: edoxaban 60 mg daily + a P2Y12 inhibitor  B: VKA + DAPT | 12 months | 1506 | Composite secondary efficacy outcome of CV death or ischemic event (including stroke, MI, ST definite, SE): HR 1.06, 95 % CI 0.71-1.69, p NR | Major or CRNM ISTH bleeding (primary safety end point): HR 0.83, 95 % CI 0.65-1.05, p=0.001 |
| [**ISAR-TRIPLE**](https://www.sciencedirect.com/science/article/pii/S0735109715008013?via%3Dihub) | A: VKA + 75 to 200 mg aspirin + 6 week clopodogrel therapy  B: VKA + 75 to 200 mg aspirin + 6 month clopidogrel therapy | 9 months | 614 | Composite of death, myocardial infarction (MI), definite ST, stroke, or TIMI major bleeding at 9 months: HR 1.14, 95 % CI 0.68-1.91, p=0.63 | TIMI major bleeding: HR 1.35, 95 % CI 0.64-2.84, p 0.44 |
| [**WOEST**](https://www.thelancet.com/journals/lancet/article/PIIS0140-6736(12)62177-1/fulltext) | A: VKA + clopidogrel  B: VKA + clopidogrel + aspirin | 12 months | 573 | Composite secondary end point of death, MI, stroke, target-vessel revascularisation, and ST: HR 0.60, 95 % CI 0.38-0.94, p=0.025 | Any bleeding episodes (primary end point): HR 0.36, 95 % CI 0.26-0.50, p < 0.0001 |

**Supplementary Table 2: Ongoing RCTs on Acute Coronary Syndromes.**

| Trial number | Trial name | Study population | Interventions | Primary outcome measures | Phase |
| --- | --- | --- | --- | --- | --- |
| NCT03331484 | CAPITAL PCI AF | ACS patients with AF (n = 40) | Ticagrelor plus rivaroxaban | Composite of TIMI bleeds | 3 |
| NCT03357874 | TROUPER | ACS patients with CKD (n = 514) | Clopidogrel vs. ticagrelor | Rate of MACE | 3 |
| NCT04718025 | ELECTRA-SIRIO | ACS patients (n = 4500) | Low-dose ticagrelor plus aspirin vs. low-dose ticagrelor plus placebo vs. standard dose ticagrelor plus aspirin | Bleeding, death from any cause, non-fatal MI, or non-fatal stroke | 3 |
| NCT05162053 | PK/PD Study of Vicagrel and Clopidogrel in healthy subjects with different CYP2C19 metabolizers | Healthy subjects with different CYP2C19 metabolizers (n = 128) | Cross-dosing of vicagrel and clopidogrel in different metabolizer groups | Inhibition of platelet aggregation, platelet reactivity, maximum plasma concentration of vicagrel and clopidogrel, AUC over a dosing interval | 1 |
| NCT05233124 | OVER-TIME | ACS patients with coronary artery ectasia (n = 60) | DAPT of aspirin plus clopidogrel vs. clopidogrel monotherapy plus low-dose rivaroxaban | Composite of cardiovascular death, recurrent MI, and repeated vascularization, composite of minor and major bleeding events | 2 |
| NCT05577988 | ADEN | ACS patients (n = 2468) | Stop aspirin for ticagrelor or prasugrel vs. aspirin or clopidogrel guided by genetic testing | Rate of combined major and minor bleeding events | 3 |
| NCT05638867 | NOAC therapy guided by PARIS Risk Score and D-dimer in patients with ACS after PCI | ACS patients with high ischaemic risk (n = 3944) | Aspirin plus clopidogrel plus rivaroxaban for 3 months followed by DAPT vs. aspirin plus clopidogrel after PCI | MACCE | 3 |
| NCT05779059 | PROTEUS | ACS patients (NSTEMI, unstable angina) (n = 50) | Initial ticagrelor and switch to prasugrel at Day 45 or initial prasugrel and switch to ticagrelor at Day 45 | Platelet reactivity | 3 |
| NCT05825573 | ARGONAUT | Patients with intra-cardiac thrombus (n = 340) | VKA vs. DOAC | Net clinical benefit | 3 |
| NCT05093790 | A study to evaluate BMS-986141 added on to aspirin or ticagrelor or the combination, on thrombus formation in a thrombosis chamber model in participants with stable coronary artery disease and healthy participants | Stable CAD (n = 55) | Ticagrelor plus BMS-986141 vs. aspirin plus BMS-986141 vs. ticagrelor plus aspirin plus BMS-986141 vs. BMS-986141 | Change from baseline in thrombus area post-treatment BMS-986141 | 2 |
| NCT05122455 | Effects of edoxaban on platelet aggregation in patients with stable CAD | Stable CAD (n = 70) | Aspirin vs. aspirin plus edoxaban, followed by clopidogrel monotherapy vs. clopidogrel plus edoxaban, followed by edoxaban monotherapy | Platelet aggregability | 2/3 |

**Supplementary Table 3: Characteristics of studies on extended anticoagulation after incident VTE.**

| **Intervention** | **Study** | **Methods** | **% of patients with PE** | **Treatment Duration (months)** | **Recurrence Proportion (% vs. comparison)** | **Recurrence Risk (HR)** |
| --- | --- | --- | --- | --- | --- | --- |
| **Apixaban** | AMPLIFY Extension, 2013 | Placebo vs. Apixaban 5/2.5 mg twice daily | 35 | 12 | Api 5: 1.7  Api 2.5: 1.7  Placebo: 8.8 | Api 5 vs. Placebo: 0.36 [0.25-0.53]  Api 2.5 vs. Placebo 0.33 [0.22-0.48] |
| **Dabigatran** | RESONATE, 2013 | Placebo vs. Dabigatran 150 mg twice daily | 33 | 6-18 | 0.4 vs. 5.6 | 0.08 [0.02-0.25] |
|  | REMEDY, 2013 | Warfarin vs. Dabigatran 150 mg twice daily | 35 | 18-36 | 1.8 vs. 1.3 | 1.44 [0.78-2.64] |
| **Rivaroxaban** | EINSTEIN Extension, 2010 | Placebo vs. Rivaroxaban 20 mg once daily | 38 | 6-12 | 1.3 vs. 7.1 | 0.18 [0.09-0.39] |
|  | EINSTEIN Choice, 2017 | Aspirin 100 mg once daily vs. Rivaroxaban 20 mg or 10 mg once daily | 49 | 12 | Riva 20: 1.5  Riva 10: 1.2  Aspirin: 4.4 | Riva 20 vs. Aspirin: 0.34 [0.2-0.59]  Riva 10 vs. Aspirin: 0.26 [0.14-0.47] |
| **Aspirin** | WARFASA, 2012 | Placebo vs. Aspirin 100 mg once daily | 40 | > 24 | 6.6 vs. 11.2 | 0.58 [0.36-0.93] |
|  | ASPIRE, 2012 | Placebo vs. Aspirin 100 mg once daily | 30 | 2-4 years | 4.8 vs. 6.5 | 0.74 [0.52-1.05] |

**Supplementary Table 4: Clinical trials comparing DOAC versus LMWH for cancer-associated VTE.**

| **RCTs** | **Methods** | **Efficacy Outcome** | **Incidence of Efficacy Outcome** | **Incidence of Major Bleeding** |
| --- | --- | --- | --- | --- |
| **ADAM VTE** | Apixaban vs. Dalteparin, 300 patients, 6 months follow up | Venous or Arterial Thromboembolism | Apixaban: 6% Dalteparin: 6% HR: 0.93 [0.43-2.02] | Apixaban: 0% Dalteparin: 1.4% HR: not estimable |
| **CARAVAGGIO** | Apixaban vs. Dalteparin, 1155 patients, 6 months follow up | Recurrent VTE | Apixaban: 5.6% Dalteparin: 7.9% HR: 0.63 [0.37-1.07] | Apixaban: 3.8% Dalteparin: 4.0% HR: 0.82 [0.40-1.69] |
| **HOKUSAI VTE Cancer** | Edoxaban vs. Dalteparinm 1050 patients, 12 months follow up | Recurrent VTE | Edoxaban: 7.9% Dalteparin:11.3% HR: 0.71 [0.48-1.06] | Edoxaban: 6.9% Dalteparin: 4.0% HR: 1.77 [1.03-3.04] |
| **SELECT-D** | Rivaroxaban vs. Dalteparin, 460 patients, 6 months follow up | Recurrent VTE | Rivaroxaban: 4% Dalteparin: 11% HR: 0.43 [0.19-0.99] | Rivaroxaban: 6% Dalteparin: 4% HR: 1.83 [0.68-4.96] |

**Supplementary Table 5: Antithrombotic Treatment indications after Structural Interventions.**

| **Antithrombotic strategies** | **TAVR** | **TMVR** | **TTVR** | **M-TEER** | **T-TEER** |
| --- | --- | --- | --- | --- | --- |
| **No pre-existing indications for OAC** | No recent coronary stent (>3months): SAPT | VKA for 3 months | VKA for 3 months | long-term SAPT | long-term  SAPT |
| **No pre-existing indications for OAC** | If recent coronary stent (<3 months): DAPT for 1-6 months followed by long-term SAPT |  |  |  |  |
| **Indications for OAC** | If no recent coronary stent (>3months)-> OAC long-term | Long-term OAC alone | Long-term OAC alone | Long-term OAC alone | Long-term OAC alone |
| **Indications for OAC** | If recent coronary stent (<3 months): SAPT for 1-6 months along with long-term OAC |  |  |  |  |

**Supplementary Table 6: Major RCTs on Antithrombotic Treatment after Cardiac Surgery.**

|  | **DACAB** | **POPular-CABG** | **Gao et al.** | **CASCADE** | **FREEDOM** | **CURE** |
| --- | --- | --- | --- | --- | --- | --- |
| **Study design** | RCT | RCT | RCT | RCT | RCT post-hoc | RCT post-hoc |
| **Patients on D/SAPT** | 168/166 | 247/249 | 124/125 | 56/57 | 544/251 | 1011/1069 |
| **D/SAPT**  **Lenght** | 12 months | 12 months | 3 months | 12 months +  8 years | 5 years | 12 months |
| **% of saphenous vein patency S/DAPT** | DAPT: 88.7  SAPT: 76.5 | DAPT: 89.5  SAPT: 90.9 | DAPT: 91.6  SAPT: 85.7 | DAPT: 94.3  SAPT: 93.2  +  DAPT: 89.1  SAPT: 91.2 | Not available | Not available |
| **MACE** | Low incidence of MACE | No clinically significant differences | No clinically significant differences | No  Clinically significant difference at 12 months +  Reduction in mortality, MACE and disease progression at 8 years | No clinically significant differences | DAPT reduces incidence of cardiovascular death, MI, and stroke |
